# Improved mini-Tn*7* Delivery Plasmids for Fluorescent Labeling of Stenotrophomonas maltophilia

**DOI:** 10.1128/aem.00317-23

**Published:** 2023-05-17

**Authors:** Uwe Mamat, Manuel Hein, Dörte Grella, Claire S. Taylor, Thomas Scholzen, Ifey Alio, Wolfgang R. Streit, Pol Huedo, Xavier Coves, Oscar Conchillo-Solé, Andromeda-Celeste Gómez, Isidre Gibert, Daniel Yero, Ulrich E. Schaible

**Affiliations:** a Cellular Microbiology, Priority Research Area Infections, Research Center Borstel, Leibniz Lung Center, Borstel, Germany; b Core Facility Fluorescence Cytometry, Research Center Borstel, Leibniz Lung Center, Borstel, Germany; c Department of Microbiology and Biotechnology, Universität Hamburg, Hamburg, Germany; d Institut de Biotecnologia i de Biomedicina (IBB), Universitat Autònoma de Barcelona (UAB), Barcelona, Spain; e Departament de Genètica i de Microbiologia, Universitat Autònoma de Barcelona (UAB), Barcelona, Spain; University of Michigan-Ann Arbor

**Keywords:** *Stenotrophomonas maltophilia*, fluorescent labeling, transposon, mini-Tn*7*, biofilm

## Abstract

Fluorescently labeled bacterial cells have become indispensable for many aspects of microbiological research, including studies on biofilm formation as an important virulence factor of various opportunistic bacteria of environmental origin such as Stenotrophomonas maltophilia. Using a Tn*7*-based genomic integration system, we report the construction of improved mini-Tn*7* delivery plasmids for labeling of S. maltophilia with sfGFP, mCherry, tdTomato and mKate2 by expressing their codon-optimized genes from a strong, constitutive promoter and an optimized ribosomal binding site. Transposition of the mini-Tn*7* transposons into single neutral sites located on average 25 nucleotides downstream of the 3′-end of the conserved *glmS* gene of different S. maltophilia wild-type strains did not have any adverse effects on the fitness of their fluorescently labeled derivatives. This was demonstrated by comparative analyses of growth, resistance profiles against 18 antibiotics of different classes, the ability to form biofilms on abiotic and biotic surfaces, also independent of the fluorescent protein expressed, and virulence in Galleria mellonella. It is also shown that the mini-Tn*7* elements remained stably integrated in the genome of S. maltophilia over a prolonged period of time in the absence of antibiotic selection pressure. Overall, we provide evidence that the new improved mini-Tn*7* delivery plasmids are valuable tools for generating fluorescently labeled S. maltophilia strains that are indistinguishable in their properties from their parental wild-type strains.

**IMPORTANCE** The bacterium S. maltophilia is an important opportunistic nosocomial pathogen that can cause bacteremia and pneumonia in immunocompromised patients with a high rate of mortality. It is now considered as a clinically relevant and notorious pathogen in cystic fibrosis patients but has also been isolated from lung specimen of healthy donors. The high intrinsic resistance to a wide range of antibiotics complicates treatment and most likely contributes to the increasing incidence of S. maltophilia infections worldwide. One important virulence-related trait of S. maltophilia is the ability to form biofilms on any surface, which may result in the development of increased transient phenotypic resistance to antimicrobials. The significance of our work is to provide a mini-Tn*7*-based labeling system for S. maltophilia to study the mechanisms of biofilm formation or host-pathogen interactions with live bacteria under non-destructive conditions.

## INTRODUCTION

Stenotrophomonas maltophilia, a non-fermentative Gram-negative bacterium of the γ-subclass of *Proteobacteria*, is found ubiquitously in nature, particularly in rhizospheric or endophytic communities, where it is implicated in different biogeochemical processes and interactions with plants (1–4). In recent years, however, the bacterium has increasingly been recognized as an emerging opportunistic nosocomial pathogen associated with a wide range of serious infections in immunocompromised patients, such as bacteremia and pneumonia with a high rate of mortality (5, 6). Although S. maltophilia has frequently been co-isolated as a predominant organism from polymicrobial infections of the cystic fibrosis (CF) lung (7), it is still under discussion whether S. maltophilia is simply a co-colonizer, an independent risk factor or, as a true co-pathogen, significantly contributes to progressive impairment of pulmonary functions in CF patients (8–10). It is well known that there is considerable genotypic and phenotypic variability among S. maltophilia isolates, which is consistent with the tremendous versatility of the bacterium to adapt to many environmental niches, including the heterogenous environment of the CF lung (11–16). The bacterium is one of the leading multidrug-resistant microorganism in the clinical setting, which makes treatment a difficult task and most likely promotes the increasing incidence of S. maltophilia infections worldwide (17, 18). It has therefore been included in the global priority list of the top 10 resistant microorganisms (TOTEM) isolated in intensive care units (19). The antimicrobial resistance (AMR) of S. maltophilia to structurally unrelated antibiotics, either encoded by the intrinsic resistome or acquired by horizontal gene transfer (20, 21), has been shown to result from efficient barrier functions of the outer membrane (22), the expression of multidrug efflux pumps (23), and the activity of a wide array of enzymes effective against antimicrobials (18). In addition, a multitude of potential virulence and virulence-associated factors, frequently expressed in a coordinated way as a function of cell density (24), have been suggested to enable the bacterium to induce inflammation, destroy infected host tissue, degrade several human serum proteins, or adhere to and colonize biotic and abiotic surfaces such as implanted medical devices (25–28). The ability of S. maltophilia to readily form biofilms on virtually any surface is a key feature of this bacterium and allows for the development of transient phenotypic resistance to antimicrobials and persistence within the host over an extended period of time (2, 18).

The availability of a large number of genome sequences and various molecular tools for genetic manipulation of S. maltophilia has not only facilitated the decoding of phylogenetic relationships between S. maltophilia isolates, but has also improved the identification and characterization of resistance determinants and virulence factors of the bacterium (2, 13, 15, 29–32). On the other hand, however, the cellular and molecular mechanisms underlying the pathogenesis of S. maltophilia infections are far from having been thoroughly investigated. These and other studies, e.g., on biofilm formation as an important factor in the pathogenesis of various opportunistic bacteria, often require the labeling of bacterial cells with bright and stable fluorescent proteins, which should preferentially be expressed by easily transferable genetic elements (33, 34). Plasmids carrying genes for fluorescent proteins undoubtedly meet the criteria of being easily transferable and giving the host bacteria increased fluorescence intensity as a consequence of the gene dosage effect. However, they can be lost in the absence of continuous selection pressure. Furthermore, the use of antibiotics to select for plasmids is rather inappropriate for studies on biofilm formation or testing of novel antibiotics. Among other mechanisms of high-level AMR development in biofilms, the self-produced extracellular polymeric substance matrix may efficiently act as a permeability barrier and thus strongly affect the penetration and efficacy of antibiotics against encased bacterial target cells (35–37). To ultimately prevent loss of fluorescence, the use of chromosomal integration systems has many advantages over plasmid-encoded fluorescent labeling of bacterial cells (34). However, for visualization of S. maltophilia cells in monospecies and mixed biofilms on abiotic and biotic surfaces, so far mainly fluorescent dyes have been used, including Syto 62 and Syto 9 fluorescent nucleic acid stains (38–40), propidium iodide (14, 41, 42), or fluorescein isothiocyanate (FITC) (43).

Here, we report the construction of improved mini-Tn*7* delivery plasmids to enable stable labeling of S. maltophilia with various fluorescent proteins through the expression of codon-optimized genes from a strong, constitutive promoter and an optimized ribosomal binding site. It is shown that the chromosomally integrated genes did neither affect bacterial growth, the antibiotic resistance pattern, biofilm formation, nor virulence of the fluorescently tagged S. maltophilia strains.

## RESULTS AND DISCUSSION

### The 3′-end of the *glmS* gene in S. maltophilia.

Mini-Tn*7* transposons have become valuable tools for inserting heterologous genes into bacterial genomes in a site- and orientation-specific manner (44–49). In the presence of the Tn*7*-encoded TnsABCD(E) transposase complex, the right (Tn*7*R) and left (Tn*7*L) ends of the transposon are sufficient for transposition of mini-Tn*7* elements at a high frequency into a single Tn*7* insertion site that is located about 25 nucleotides downstream of the conserved *glmS* gene for the essential glutamine-fructose-6-phosphate aminotransferase involved in cell wall metabolism of many bacteria (44, 50, 51). The transposase complex additionally requires an *att*Tn*7*-associated sequence motif at the immediate 3′-end of *glmS* to direct transposition into the single insertion site (50). With very few exceptions that contain multiple *glmS* genes and *att*Tn*7* sites, such as members of the genus *Burkholderia* (52), most bacterial species carry only a single *glmS* gene and thus only one *att*Tn*7* site, which allows the insertion of a single-copy gene at a unique neutral intergenic site of the bacterial genome.

To the best of our knowledge, there is a very limited number of previous studies using S. maltophilia strains that have been fluorescently labeled with a mini-Tn*7* delivery system (53–55). In fact, the plasmid pURR25 carrying the mini-Tn*7*KSGFP transposon and engineered for use in other γ-*Proteobacteria*, such as Photorhabdus luminescens, Pseudomonas aeruginosa or *Serratia* spp. (56), was applied in all these studies for labeling of S. maltophilia with GFP, but without providing further details or background information.

To address the question of whether S. maltophilia is indeed a suitable host for the development of a site-specific chromosome integration system based on Tn*7*, we searched the NCBI genome database and found only one *glmS* gene in most S. maltophilia strains, which suggests a single *att*Tn*7* site in this bacterium. A total of 75 S. maltophilia strains, belonging to 15 out of 23 different monophyletic lineages (13), was then randomly selected to compare the 3′-ends and 40 nucleotides of the intergenic regions downstream of the *glmS* gene (Fig. S1). Noteworthy, the sequence variations downstream of the *glmS* gene allowed classification of the intergenic regions into groups that were largely consistent with the monophyletic lineages of the strains, suggesting, like in many other bacteria (57, 58), a naturally evolved region as an insertion site of mini-Tn*7* transposons in S. maltophilia. This assumption was additionally supported by the fact that the *att*Tn*7*-associated sequence motif at the immediate 3′-end of *glmS* was as highly conserved in S. maltophilia as in other Gram-negative bacteria. Taken together, the data described above strongly suggested that S. maltophilia contains the genetic background required for single-copy insertion of Tn*7*-based genetic elements.

### Construction of mini-Tn*7* delivery plasmids for S. maltophilia.

It has long been known that transposable genetic elements based on Tn*7* have an extraordinarily broad host range, with at least 20 different bacterial species in which Tn*7* is able to transpose (44). The original classification of S. maltophilia as a member of the genus Pseudomonas (59), followed by assignment of the bacterium to the genus *Xanthomonas* (60) and reclassification as *Stenotrophomonas* (61) has prompted us to first test the utility of pUC18T-mini-Tn*7*T-Gm-*ecfp*, pUC18T-mini-Tn*7*T-Gm-*eyfp* and pUC18T-mini-Tn*7*T-Gm-*dsRedExpress* for fluorescent labeling of S. maltophilia. The latter mobilizable mini-Tn*7* delivery vectors, which have been successfully developed to fluorescently label P. aeruginosa (45), were transferred from E. coli DH5α donor strains to different S. maltophilia recipients by four-parental mating experiments, using E. coli DH5α with pRK2013 for mobilization of mini-Tn*7* plasmids and E. coli SM10 (λ*pir*) carrying the plasmid pUX-BF13 for TnsABCDE transposase expression as helper strains. However, although colony PCRs using the primer pairs P_Tn_*_7_*_L_/PSmlt_glmS-up_ and PSmlt_glmS-down_/P_Tn_*_7_*_R_ indicated that insertion of the mini-Tn*7* elements occured at *att*Tn*7* in S. maltophilia, the signals from all fluorescent proteins proved to be quite weak (data not shown).

We therefore decided to develop improved mini-Tn*7* delivery plasmids derived from the base vectors pUC18R6K-mini-Tn7T-Gm and pUC18T-mini-Tn7T (44, 45). Using a multiple-step cloning strategy, the fluorescence intensity of S. maltophilia cells as examined by confocal laser-scanning microscopy (CLSM) was successively increased in the course of plasmid construction. As described in detail in Materials and Methods and depicted in Fig. S2, amplification of the fluorescence signal was mainly achieved by optimization of three functional mini-Tn*7* elements, i.e., i) adaptation of codon usage of the genes for the fluorescent proteins sfGFP, mCherry, tdTomato and mKate2 to their expression in bacteria of the family *Xanthomonadaceae*, ii) change to the strong, constitutive promoter *P_c_* from class III integron of Delftia acidovorans C17 (62, 63) to drive expression of the fluorescent proteins, and iii) replacement of the putative ribosomal site sequence GCGAGC of the *rpoD* gene of S. maltophilia K279a with the six-base consensus sequence AGGAGG seven bases upstream of the AUG start codons. Interestingly, the *P_c_* promoter was also recently used to achieve high expression levels of fluorescent proteins on mini-Tn*7* elements in P. fluorescens (64) and could therefore serve as an alternative to other constitutive promoters used for protein expression in different Gram-negative bacteria. If required, genes for other fluorescent proteins can be placed under the control of the *P_c_* promoter by replacement of the existing genes using *Bam*HI/*Sac*l cloning (see Fig. S2).

Gentamycin is one of the few antibiotics to which multidrug-resistant S. maltophilia strains are sensitive to varying degree. Therefore, we did not replace the original *aacC1* gene for aminoglycoside-(3)-*N*-acetyltransferase in the improved mini-Tn*7* elements, but usually checked the intrinsic resistance to gentamicin of the strains to be labeled before each experiment. For selection, we routinely used a concentration of 60 μg/mL gentamicin, but this could be adjusted if intrinsic resistance to this antibiotic was elevated in certain strains. As part of the initial mini-Tn*7*T-Gm transposon encoded by pUC18R6K-mini-Tn7T-Gm (Fig. S2), the *aacC1* gene was flanked by FLP recombinase target (*FRT*) sites and could therefore offer the opportunity of being removed from the genome by Flp-mediated excision to yield strains with unmarked mini-Tn*7* insertions (44). For expression of the yeast FLP recombinase, the helper plasmid pFLP2 has been widely used (45, 65). However, due to the highly intrinsic resistance of S. maltophilia to ß-lactams, the plasmid was unsuitable for removal of the FRT cassette from these bacteria. Following the approach to construct an alternative for pFLP2 in phytopathogenic *Xanthomonas* spp. (47), we cloned a 5.1-kb *Pst*l fragment containing the *cI_857_-FLP-sacB* fragment from pFLP2 into the *Pst*l site of pBBR1MCS-1, a cloning vector with moderate copy number coding for chloramphenicol resistance (66). However, various attempts to excise the *aacC1* gene in the presence of the pBBR1MCS-1-FLP2 helper plasmid failed (data not shown) and were therefore not pursued further.

### Chromosomal insertion of mini-Tn*7* elements and fluorescent labeling of S. maltophilia.

**FIG 1:**
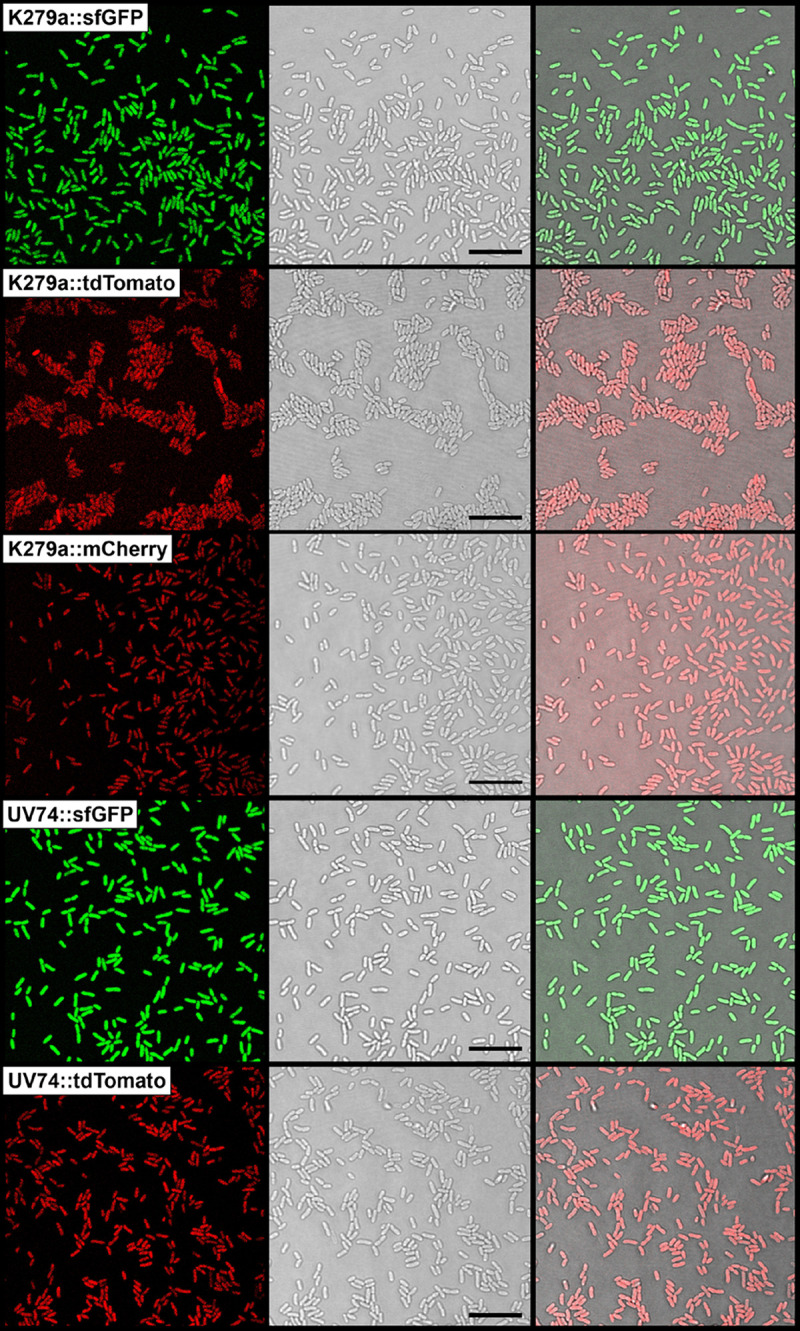
Microscopic analysis of S. maltophilia strains carrying chromosomally integrated mini-Tn*7* elements for expression of different fluorescent proteins. Confocal (left panel), transmitted light (middle panel) and merged (right panel) images of S. maltophilia strains K279a::sfGFP, K279a::tdTomato, K279a::mCherry, UV74::sfGFP and UV74::tdTomato show that the entire population of each strain is fluorescently labeled. Scale bars correspond to 10 μm.

**FIG 2:**
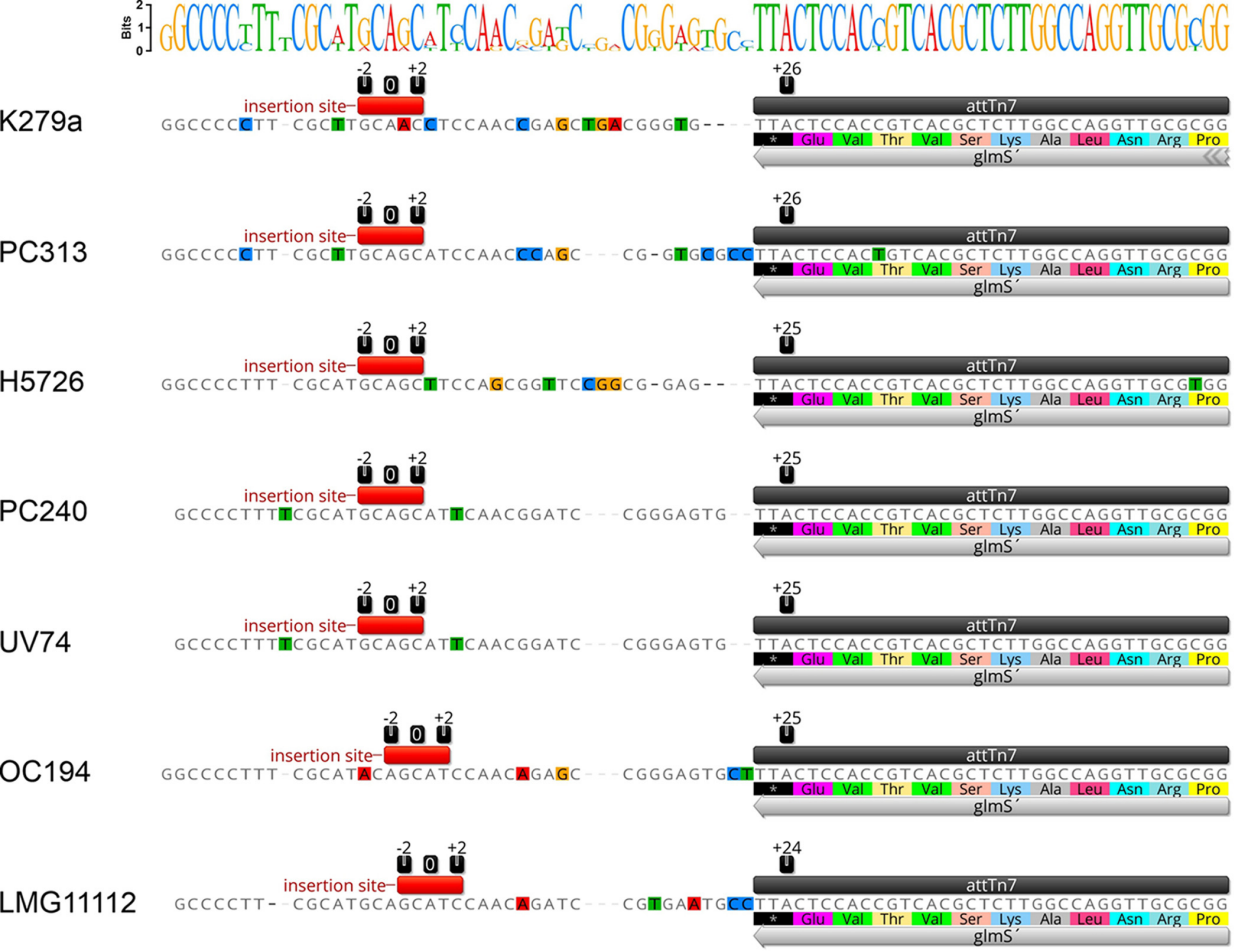
Insertion sites of mini-Tn*7* elements in various S. maltophilia strains. Alignment of the highly conserved *att*Tn*7*-associated sequence motif at the immediate 3′-end of *glmS* and downstream sequences of the intergenic region. The mini-Tn*7* insertion sites with an average distance of 25 nucleotides from the 3′-end of the *glmS* gene are indicated with a red bar. The center of the 5-bp target site is designated nucleotide position 0, while sequences in the direction of the *glmS* gene have positive numbering and nucleotides in the other direction, away from *glmS*, have negative numbering (58, 68). The alignment was created with Geneious Prime 2023.0.4 (www.geneious.com).

The improved mini-Tn*7* delivery plasmids could be transferred with high efficiency into various S. maltophilia isolates by conjugative transfer. For fluorescent labeling, we aimed not only to use a selection of strains from different monophyletic lineages but also to place focus on clinical isolates from different sources such as blood, lung puncture, vascular ulcer, perineum, oropharynx, hematologic neoplasia, or cystic fibrosis patients. As shown in Fig. 1 and Fig. S3, the four-parental mating experiments yielded fluorescently labeled strains with a high signal-to-noise ratio at single-cell resolution. While the site- and orientation-specific insertion of the mini-Tn*7* elements downstream of *glmS* were routinely verified by PCR with primer pairs P_Tn_*_7_*_L_/PSmlt_glmS-up_ and PSmlt_glmS-down_/P_Tn_*_7_*_R_, the actual mini-Tn*7* insertion sites in S. maltophilia strains K279a, PC313, H5726, PC240, UV74, OC194 and LMG11112 were determined by sequencing of the flanking mini-Tn*7* regions using DNA fragments amplified from fluorescently labeled derivatives of the strains. Because the Tn*7* transposition event generates characteristic 5-bp duplications of the insertion site (67), the host target sequence of the mini-Tn*7* elements could be readily identified in the different S. maltophilia strains (Fig. 2, Fig. S1 and S4B). According to the nomenclature of Craig and colleagues (58, 68), in which the base pair located in the middle of the 5-bp sequence is referred to as nucleotide position 0, with sequences toward *glmS* possessing a positive numbering, the average distance between the mini-Tn*7* insertion site and the 3′-end of the *glmS* gene was 25 nucleotides in S. maltophilia (Fig. 2 and Fig. S1), consistent with the distance determined in other bacteria, such as *Xanthomonas* spp. (47), P. aeruginosa (44, 45), E. coli (68), Acinetobacter baumannii (48), P. putida, Yersinia pestis, or Burkholderia thailandensis (44). Given the degree of sequence conservation of the intergenic regions downstream of the *glmS* gene within individual monophyletic lineages, it is reasonable to assume that S. maltophilia strains of the same monophyletic lineage have identical target sequences that serve as insertion sites for mini-Tn*7* transposons (Fig. S1).

### Stability of mini-Tn*7* insertions.

To answer the question of whether integration of the mini-Tn*7* transposons into the chromosome of S. maltophilia affects strain fitness, we used fluorescent derivatives of strains K279a and UV74 throughout this study. The blood isolate K279a is considered the clinical reference strain for which the first whole-genome sequence of an S. maltophilia bacterium was available (69), whereas strain UV74 isolated from a vascular ulcer was found to be genetically very similar to strain D457 (70), a bronchial-aspiration isolate that is used as a model strain for studying the expression and regulation of resistance determinants in S. maltophilia (71).

As a first step, we aimed to investigate the chromosomal stability of improved mini-Tn*7* elements in strains K279a::sfGFP, K279a::tdTomato, UV74::sfGFP and UV74::tdTomato. Since the *aacC1* gene was genetically linked to the corresponding gene for a fluorescent protein on the inserted mini-Tn*7* transposon, we considered a stable inheritance of the gentamicin resistance marker as a measure of the stability of the entire mini-Tn*7* element, including a stable replication of the gene for the fluorescence protein in the absence of continued antibiotic selection. We therefore grew all strains in media without gentamicin for five consecutive days, followed by the testing of 100 individual clones of each strain for their ability to grow in media containing the antibiotic. As expected from chromosomally inserted markers, 100% of the clones retained their mini-Tn*7*-encoded gentamicin resistance in the absence of sustained selection pressure. This result was confirmed by the detection of mini-Tn*7* elements in the correct orientation as 502-bp PCR products in 10 randomly selected clones of each strain (Fig. S4). Taken together, these data indicated that the mini-Tn*7* transposons are stable in S. maltophilia over a longer time period without the need for continuous selection pressure.

### Growth properties of fluorescently labeled S. maltophilia strains.

**FIG 3:**
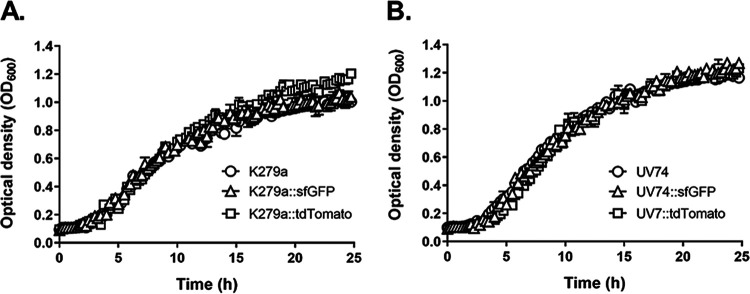
Comparative growth analysis of S. maltophilia wild-type strains and their fluorescently labeled derivatives. The growth of strains K279a, K279a::sfGFP and K279a::tdTomato (A) and UV74, UV74::sfGFP and UV74::tdTomato (B) was recorded by measuring the optical density of the cultures at 600 nm (OD_600_) at 37°C over a period of 24 h. The data are represented as means ± SD of three replicates per strain.

Acquisition of antibiotic resistance determinants, e.g., through mobile genetic elements, may be associated with fitness deficits due to an overall metabolic burden and, as a result, may lead to lower bacterial growth rates (72–74). While integration of unmarked mini-Tn*7* transposons at a unique neutral intergenic site downstream of the *glmS* gene does not appear to have adverse effects on bacterial fitness (44), we wondered whether the acquisition of the *aacC1* gene together with the mini-Tn*7* element and the development of resistance to gentamicin might affect the fitness of fluorescent S. maltophilia strains. As shown herein for S. maltophilia strains K279a::sfGFP, K279a::tdTomato, UV74::sfGFP and UV74::tdTomato, there were no growth differences between the parental strains and their fluorescent derivatives (Fig. 3). The mean generation times (mean ± SD) of K279a, K279a::sfGFP, K279a::tdTomato, UV74, UV74::sfGFP and UV74::tdTomato were similar and amounted to 106.90 ± 0.44 min, 111.90 ± 0.10 min, 111.75 ± 1.19 min, 91.22 ± 0.97 min, 93.49 ± 9.14 min and 100.37 ± 12.96 min, respectively.

To address the question of whether integrated mini-Tn*7* elements have an impact on the antibiograms of fluorescently labeled S. maltophilia in comparison to their parental strains, we examined the resistance profiles of K279a, K279a::sfGFP, K279a::tdTomato, UV74, UV74::sfGFP and UV74::tdTomato with 18 antibiotics of different classes. As shown in Table 1, the MIC values of almost all tested antibiotics against the parental strains and their fluorescently labeled derivatives were identical. Only the MIC of the combination of the antibiotic ticarcillin with the ß-lactamase inhibitor clavulanic acid against the tdTomato-labeled derivative of strain K279a was reduced by half and, conversely, the MIC values of ticarcillin-clavulanic acid combination and another combinatorial antibiotic, piperacillin-tazobactam, were doubled in UV74::tdTomato compared to UV74 and UV74::sfGFP. It must be emphasized, however, that a 2-fold dilution difference is within the intrinsic error of the broth microdilution method and is not usually considered significant in susceptibility tests (75). Overall, the results allowed us to draw the conclusion that integration of the improved mini-Tn*7* transposons does neither alter the growth characteristics nor the resistance patterns of the fluorescently labeled S. maltophilia strains.

**TABLE 1:** Antibiotic resistance profiles of S. maltophilia wild-type strains K279a and UV74 in comparison to their fluorescently labeled derivatives

Antibiotic	MIC (μg/mL)					
	K279a	K279a::sfGFP	K279a::tdTomato	UV74	UV74::sfGFP	UV74::tdTomato
Sulfonamides						
Trimethoprim-sulfamethoxazole	0.25	0.25	0.25	0.25	0.25	0.25
Polypeptides						
Colistin	2	2	2	32	32	32
Carbapenems (ß-lactam)						
Imipenem	64	64	64	256	256	256
Meropenem	8	8	8	2	2	2
Cephalosporins (ß-lactam)						
Ceftazidime	4	4	4	2	2	2
Penicillins (ß-lactam)						
Piperacillin-tazobactam	16	16	16	64	64	128
Ticarcillin-clavulanic acid	2	2	1	16	16	32
Aminoglycosides						
Tobramycin	32	32	32	8	8	8
Amikacin	16	16	16	16	16	16
Streptomycin	64	64	64	64	64	64
Chloramphenicol						
Chloramphenicol	16	16	16	16	16	16
Tetracyclines						
Tetracycline	16	16	16	8	8	8
Minocycline	0.5	0.5	0.5	0.25	0.25	0.25
Tigecycline	2	2	2	2	2	2
Fluoroquinolones						
Ciprofloxacin	8	8	8	16	16	16
Levofloxacin	4	4	4	1	1	1
Ofloxacin	4	4	4	2	2	2
Norfloxacin	32	32	32	128	128	128

### Biofilm formation of fluorescently labeled S. maltophilia strains.

**FIG 4:**
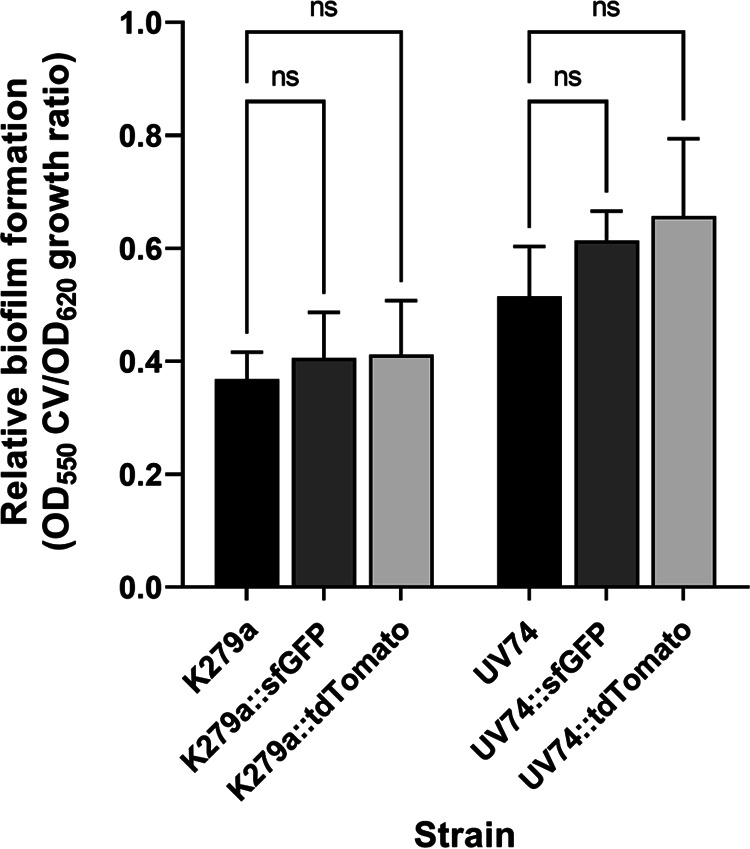
Biofilm formation of S. maltophilia wild-type strains K279a and UV74 in comparison to their sfGFP- and tdTomato-labeled derivatives. The amount of each biofilm as quantified by crystal violet staining (OD_550_ value) was normalized by the optical density of the cell suspension at 620 nm (OD_620_) and shown as relative biofilm formation. Values are presented as means ± SD of six technical replicates for each of four biological replicates per strain. Statistical significance was determined by the non-parametric Kruskal-Wallis one-way Analysis of Variance (ANOVA) test corrected for multiple comparisons using Dunn’s test (ns: not significant).

The ability to form biofilms as an important virulence factor is a characteristic feature of S. maltophilia (15, 41, 76–78). Because biofilm growth is another measure of bacterial fitness, changes in the capacity to form biofilms can have far-reaching consequences, such as altering the pathogenic and resistance potential of bacteria (74). To determine whether fluorescently labeled S. maltophilia strains are unchanged in their ability to produce biofilm, we first used crystal violet (CV) staining to quantify the amount of biofilm relative to bacterial growth to deduce the values of relative biofilm formation for strains K279a, K279a::sfGFP, K279a::tdTomato, UV74, UV74::sfGFP and UV74::tdTomato (Fig. 4). Based on the mean ratio (OD_550_ of CV/OD_620_ of cell growth), we were able to confirm that strains with a diffusible signal factor-based quorum sensing system of the *rpf*-2 type (UV74) are stronger biofilm formers than strains of the *rpf*-1 type (K279a) (78). Our results further showed only marginal, statistically non-significant differences in biofilm formation between the parent strains and their fluorescently labeled derivatives under the experimental conditions used, suggesting once again that the chromosomally integrated mini-Tn*7* elements did not negatively affect the fitness of the bacterial cells.

**FIG 5:**
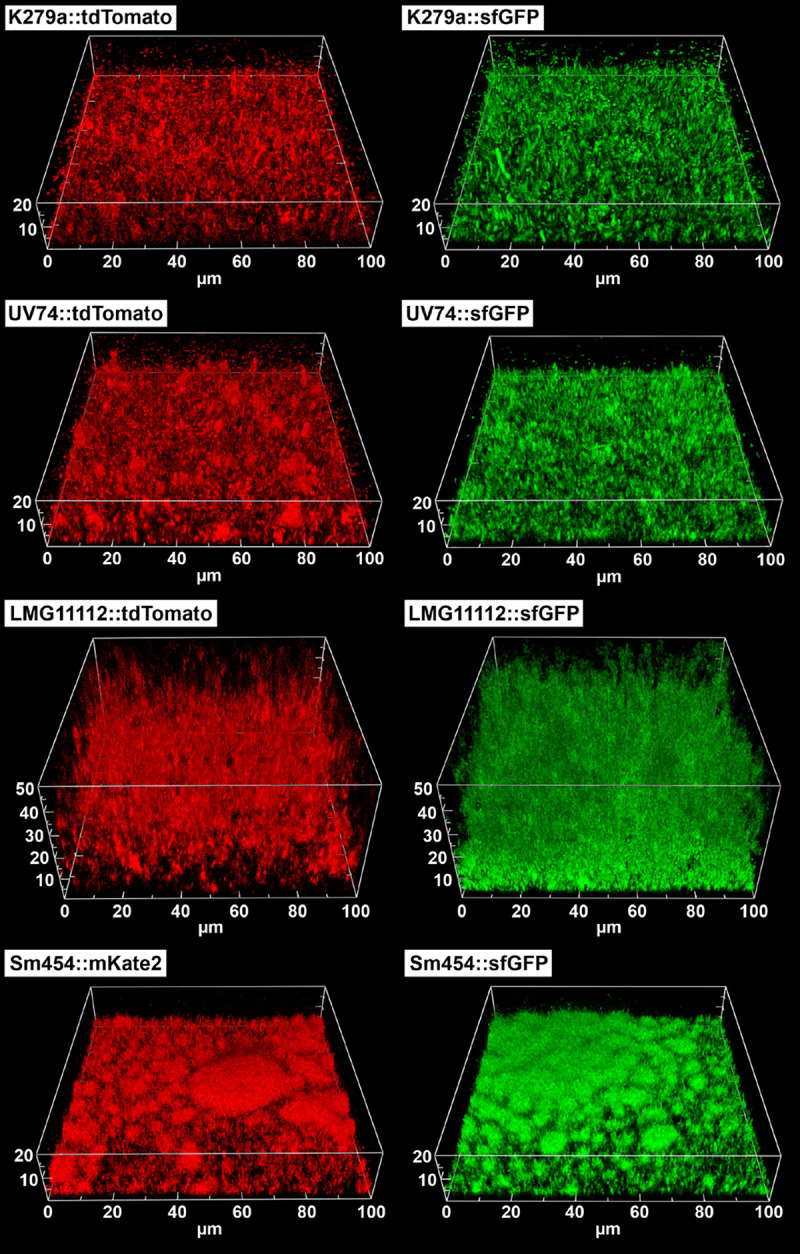
Biofilm formation of fluorescently labeled S. maltophilia strains as analyzed by confocal laser-scanning microscopy (CLSM). Representative CLSM images show the comparison of biofilm architecture of strains expressing two different fluorescent proteins. Biofilms composed of sfGFP- and mKate2-labeled bacterial cells were grown on a polymer surface for 72 h, whereas biofilms consisting of tdTomato-labeled cells were grown for 48 h under static conditions. The three-dimensional images were generated with *daime* (93).

Imaging techniques are increasingly being used in biofilm research, with bacteria expressing fluorescent proteins finding wide application, for example, in studies of spatial biofilm structure or the dynamics of biofilm maturation as a function of time (79, 80). Here we used confocal laser-scanning microscopy (CLSM) to visualize the three-dimensional architecture of biofilms formed by fluorescently labeled S. maltophilia strains. For this purpose, the biofilms were grown on a polymer surface under static conditions for either 48 h (bacteria with tdTomato labeling) or 72 h (bacteria with sfGFP or mKate2 labeling), followed by reconstruction of representative biofilm images from CLSM Z-axis serial sections (Fig. 5 and Fig. S5). As expected, the CLSM images showed biofilm-forming ability of all strains on a solid polymeric surface and strain-dependent variations in biofilm architecture, characterized by loosely to densely packed populations of bacterial cells. All strains tested appeared to maintain the biofilm mass formed after 48 h throughout the 72-h experimental period, with strain S. maltophilia LMG11112, a clinical isolate from a lung puncture, proving to be the most potent biofilm producer in this study after 48 and 72 h of growth (Fig. 5). However, we attached even more importance to the observation that the various fluorescent proteins encoded on the corresponding mini-Tn*7* transposons have no effect on biofilm architecture. This was particularly evident in the Sm454::sfGFP and Sm454::mKate2 strains, which had formed biofilms of microcolonies of varying sizes after 72 h of incubation (Fig. 5 and Fig. S6). Interestingly, and as the example of strain Sm454::sfGFP showed (Fig. S7), the microcolonies possessed a characteristic ultrastructure consisting of densely packed cell layers adhering to the polymer surface and layers with loosely packed cell aggregates. The latter were reminiscent of rosettes, which appeared to be formed by adhesion between cells at their poles. Of note, a similar rosette-like biofilm architecture of S. maltophilia Sm454 was recently observed, but visualized using a live/dead staining protocol (41). However, the mechanisms responsible for intercellular cohesion still needs to be elucidated, e.g., whether the aggregates are held together by a unipolar adhesion polysaccharide, similar to that described for rosette formation of cells in biofilms of other bacteria, such as Rhodopseudomonas palustris (81) or Agrobacterium tumefaciens (82). Taken together, the results indicated that the presence of the mini-Tn*7* elements does not affect the biofilm formation of the fluorescently labeled strains compared with their parental strains, nor does it lead to differences between biofilms of differently labeled cells of the same strain. The differently labeled S. maltophilia strains would thus be interchangeable if necessary.

**FIG 6:**
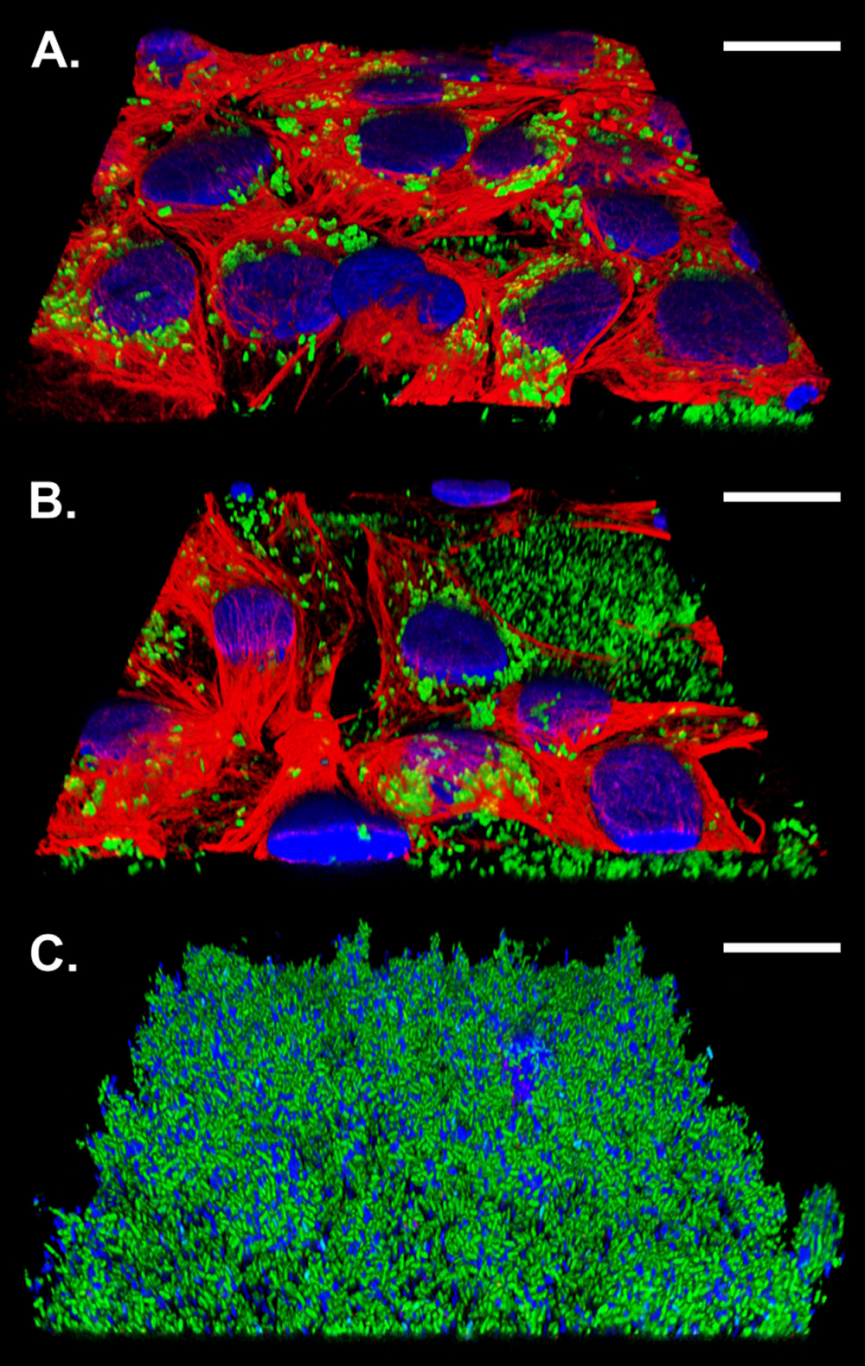
Biofilm formation of S. maltophilia Sm314::sfGFP on adherent Calu-3 cells. The representative CLSM images show the succession of biofilm formation 16 h (A), 24 h (B) and 48 h (C) after infection. The bacterial cells glow with green fluorescence, the nuclei are stained blue with DAPI, and the Alexa Fluor 647 conjugated C-11 monoclonal antibody reacts with a conserved epitope of human cytokeratins 4, 5, 6, 8, 10, 13 and 18 (red). The confocal image stacks were processed with Leica's Lightning Deconvolution Tool (Leica Application Suite X, version 3.0.0_15697) and the Imaris Viewer 10.0.0 (Oxford Instruments). Each scale bar represents 20 μm.

Using CLSM, we were also able to monitor the succession of biofilm formation of S. maltophilia Sm314::sfGFP on confluent Calu-3 cell monolayers over a 48-h period (Fig. 6). The gradual damage of Calu-3 cells by the growing biofilm, starting with the formation of aggregates of bacterial cells on the Calu-3 cells after a period of 16 h, followed by the onset of biofilm formation on the polymer surface after 24 h, and finally ending with the complete destruction of the Calu-3 monolayer after 48 h, confirmed the role of biofilm as one of the major virulence determinants in pathogen-host interaction between S. maltophilia and pulmonary epithelial cells. Collectively, our results provided evidence that the fluorescently labeled S. maltophilia strains are equally capable of forming biofilms on abiotic and biotic surfaces, suggesting that they could serve as valuable tools in biofilm research or investigations into the host-pathogen interactions.

### Virulence of fluorescently labeled S. maltophilia strains.

**FIG 7:**
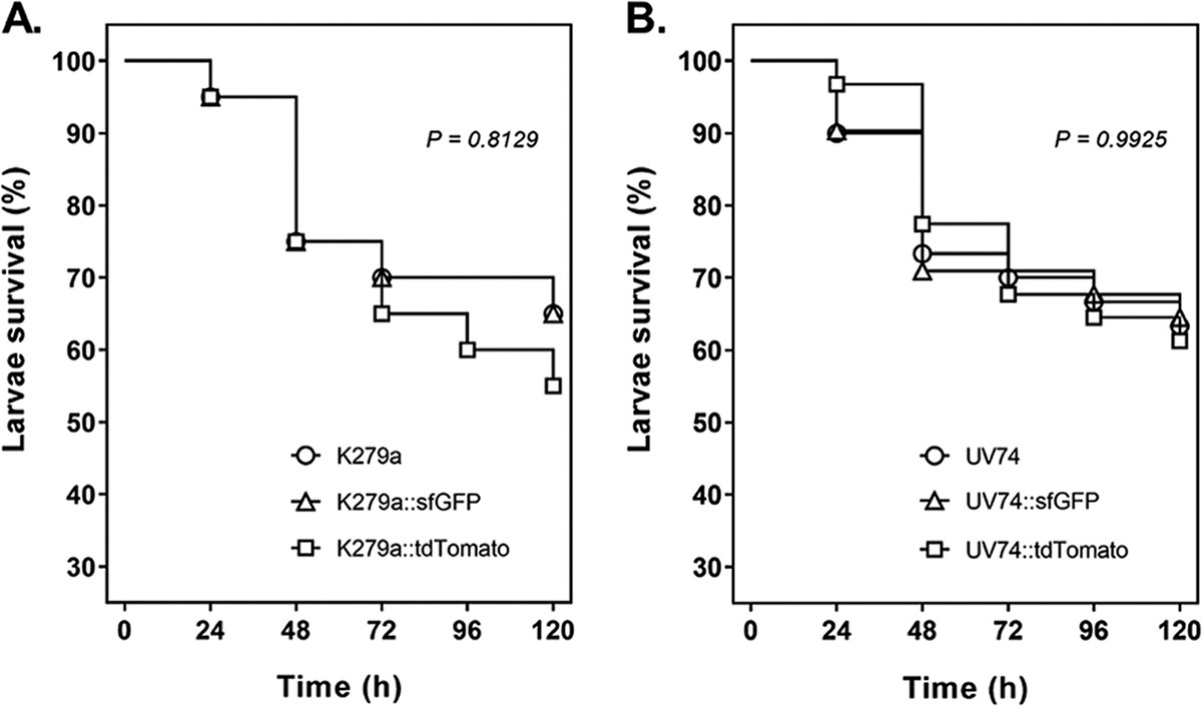
Virulence of S. maltophilia wild-type strains in comparison to their fluorescently labeled derivatives in G. mellonella larvae. Survival of infected larvae (5 × 10^5^ CFU of each strain per larva) was examined for strains K279a, K279a::sfGFP, and K279a::tdTomato (A) and UV74, UV74::sfGFP, and UV74::tdTomato (B) at 24-h intervals over 120 h, and survival curves were analyzed using the Kaplan-Meier method (log-rank test: not significant, *P > *0.05).

Comparative virulence tests using the larvae of the greater wax moth Galleria mellonella were performed with the strains K279a and UV74 and their sfGFP- and tdTomato-labeled derivatives. As Fig. 7 shows, all strains were moderately virulent in the larval killing assays, with no significant difference between wild-type and fluorescently labeled strains. The inoculum used, approximately 5 × 10^5^ CFU, resulted in 5–10% mortality for all strains 24 h postinfection, while 35–45% of larvae were killed after 120 h. However, this assay was only useful for demonstrating that fluorescent labeling has no effect on the virulence of the strains. As shown previously, fluorescently labeled strains are not suitable for monitoring the course of infection within the larvae due to their autofluorescence and the fact that the cuticle does not allow detection of fluorescence inside the larvae (83).

## MATERIALS AND METHODS

### Bacterial strains, plasmids and growth conditions.

All strains and plasmids used in the present study are described in Table 2. Unless otherwise stated, the E. coli and S. maltophilia strains were grown aerobically with shaking (220 rpm) at 37°C in LB-Miller medium (10 g of tryptone, 5 g of yeast extract, 10 g of NaCl per L), except E. coli SM10 (λ*pir*)/pUX-BF13 was routinely cultured at 30°C. For plasmids containing the R6Kγ origin of replication, E. coli SY327 expressing the λ PI protein served as a cloning host. All other plasmids were constructed and propagated in E. coli DH5α. Gentamycin at concentrations of 15 μg/mL, 30 μg/mL and 60 μg/mL, kanamycin (30 μg/mL), ampicillin (100 μg/mL), or norfloxacin (5 μg/mL) were added to the media as required.

**TABLE 2:** Bacterial strains and plasmids used in this work

Strain or plasmid	Genotype or description	Source or reference
Escherichia coli		
SM10 (λ*pir*)	Km^R^ *thi-1 thr leu tonA lacY supE recA*::RP4-2-Tc::Mu λ*pir*	(88, 89)
SY327	Δ(*lac pro*) *argE*(*Am*) *recA56 rif ^R^ nalA* λ *pir*	(88)
DH5α	F^–^ Φ80*lacZ*ΔM15 Δ(*lacZYA*-*argF*) U169 *recA1 endA1 hsdR17*(r_K_^–^m_K_^+^) *phoA supE44 thi-1 gyrA96 relA1* λ^–^	(97)
Stenotrophomonas maltophilia		
K279a	Clinical isolate from the blood of a cancer patient	(98)
LMG11112	Clinical isolate from lung puncture	Belgian Co-ordinated Collections of Microorganisms (BCCM)
UV74	Clinical isolate from vascular ulcer	(95)
PC313	Clinical isolate from perineum	(95)
PC240	Clinical isolate from perineum	(95)
OC194	Clinical isolate from oropharynx	(95)
H5726	Clinical isolate from hematologic neoplasia	(95)
Sm454	Clinical isolate from a cystic fibrosis patient	(15)
Sm314	Clinical isolate from a cystic fibrosis patient	(15)
K279a::sfGFP	K279a carrying the mini-Tn*7*T-Gm-Pc-sfGFP_opt element downstream of the *glmS* (SMLT_RS19505) gene	This study
K279a::mCherry	K279a carrying the mini-Tn*7*T-Gm-Pc-mCherry_opt element downstream of the *glmS* (SMLT_RS19505) gene	This study
K279a::tdTomato	K279a carrying the mini-Tn*7*T-Gm-Pc-tdTomato_opt element downstream of the *glmS* (SMLT_RS19505) gene	This study
LMG11112::sfGFP	LMG11112 carrying the mini-Tn*7*T-Gm-Pc-sfGFP_opt element downstream of the *glmS* gene	This study
LMG11112::tdTomato	LMG11112 carrying the mini-Tn*7*T-Gm-Pc-tdTomato_opt element downstream of the *glmS* gene	This study
UV74::sfGFP	UV74 carrying the mini-Tn*7*T-Gm-Pc-sfGFP_opt element downstream of the *glmS* gene	This study
UV74::tdTomato	UV74 carrying the mini-Tn*7*T-Gm-Pc-tdTomato_opt element downstream of the *glmS* gene	This study
PC313::sfGFP	PC313 carrying the mini-Tn*7*T-Gm-Pc-sfGFP_opt element downstream of the *glmS* gene	This study
PC313::tdTomato	PC313 carrying the mini-Tn*7*T-Gm-Pc-tdTomato_opt element downstream of the *glmS* gene	This study
PC240::sfGFP	PC240 carrying the mini-Tn*7*T-Gm-Pc-sfGFP_opt element downstream of the *glmS* gene	This study
PC240::tdTomato	PC240 carrying the mini-Tn*7*T-Gm-Pc-tdTomato_opt element downstream of the *glmS* gene	This study
OC194::sfGFP	OC194 carrying the mini-Tn*7*T-Gm-Pc-sfGFP_opt element downstream of the *glmS* gene	This study
OC194::tdTomato	OC194 carrying the mini-Tn*7*T-Gm-Pc-tdTomato_opt element downstream of the *glmS* gene	This study
H5726::sfGFP	H5726 carrying the mini-Tn*7*T-Gm-Pc-sfGFP_opt element downstream of the *glmS* gene	This study
Sm454::sfGFP	Sm454 carrying the mini-Tn*7*T-Gm-Pc-sfGFP_opt element downstream of the *glmS* gene	This study
Sm454::mKate2	Sm454 carrying the mini-Tn*7*T-Gm-Pc-mKate2_opt element downstream of the *glmS* gene	This study
Sm314::sfGFP	Sm314 carrying the mini-Tn*7*T-Gm-Pc-sfGFP_opt element downstream of the *glmS* gene	This study
Plasmids		
pRK2013	Kan^R^; RK2-derived helper plasmid carrying the *tra* and *mob* genes for mobilization of plasmids containing *oriT*	(87)
pUC18R6K-mini-Tn7T-Gm	Amp^R^; Gm^R^ on mini-Tn*7*; R6Kγ-based mini-Tn*7* cloning and integration vector	(44, 45) (Addgene plasmid # 65022; http://n2t.net/addgene:65022; RRID:Addgene_65022)
pUC18T-mini-Tn7T	Amp^R^; mobilizable mini-Tn*7* cloning and integration vector	(44, 45) (Addgene plasmid # 64957; http://n2t.net/addgene:64957; RRID:Addgene_64957)
pUX-BF13	Amp^R^; R6Kγ-based helper plasmid containing the Tn*7* transposase genes *tnsABCDE* for transposition of mini-Tn*7* elements	(90)
pGEM-T Easy-mCherry	Amp^R^; pGEM-T Easy vector (Promega) carrying the gene for the mCherry red fluorescent protein	Anke Becker, Center for Synthetic Microbiology (SYNMIKRO), Marburg, Germany
pEX-A2-mCherry_opt	Amp^R^; pEX-A2 vector with a synthetic 779-bp *Apa*I/*Bam*HI insert containing an mCherry gene with optimized codon usage for *Xanthomonadaceae* and fused with an optimized ribosomal binding site of the *rpoD* (SMLT_RS19820) gene of K279a	Eurofins Genomics, Ebersberg, Germany; this study
pEX-A2-sfGFP_opt	Amp^R^; pEX-A2 vector with a synthetic 788-bp *Apa*I/*Bam*HI insert containing an sfGFP gene with optimized codon usage for *Xanthomonadaceae* and fused with an optimized ribosomal binding site of the *rpoD* (SMLT_RS19820) gene of K279a	Eurofins Genomics, Ebersberg, Germany; this study
pEX-A128-Pc	Amp^R^; pEX-A128 vector with a synthetic 237-bp *Kpn*I/*Bam*HI insert containing the P_c_ promoter from class III integron of Delftia acidovorans C17	Eurofins Genomics, Ebersberg, Germany; this study
pUC18R6K-mini-Tn7T-Gm- rpoD	Amp^R^; Gm^R^ on mini-Tn*7*; pUC18R6K-mini-Tn*7*T-Gm with a 224-bp *Kpn*I/*Xho*I insert containing the promoter and ribosomal binding site of the *rpoD* (SMLT_RS19820) gene of K279a	This study
pUC18R6K-mini-Tn7T-Gm-rpoD-mCherry	Amp^R^; Gm^R^ on mini-Tn*7*; pUC18R6K-mini-Tn*7*T-Gm-rpoD with a 723-bp *Xho*I/*Bam*HI insert containing the mCherry gene of pGEM-T Easy-mCherry	This study
pUC18T-mini-Tn7T-Gm-rpoD-mCherry	Amp^R^; Gm^R^ on mini-Tn*7*; hybrid plasmid comprising the 3104-bp and 2370-bp *Kpn*I/*Sal*I fragments of pUC18T-mini-Tn*7*T and pUC18R6K-mini-Tn*7*T-Gm-rpoD-mCherry, respectively	This study
pUC18T-mini-Tn7T-Gm-rpoD-mCherry_opt	Amp^R^; Gm^R^ on mini-Tn*7*; hybrid plasmid comprising the 4700-bp and 779-bp *Apa*I/*Bam*HI fragments of pUC18T-mini-Tn*7*T-Gm-rpoD-mCherry and pEX-A2-mCherry_opt, respectively	This study
pUC18T-mini-Tn7T-Gm-rpoD-sfGFP_opt	Amp^R^; Gm^R^ on mini-Tn*7*; hybrid plasmid comprising the 4700-bp and 788-bp *Apa*I/*Bam*HI fragments of pUC18T-mini-Tn*7*T-Gm-rpoD-mCherry and pEX-A2-sfGFP_opt, respectively	This study
pUC18T-mini-Tn7T-Gm-Pc	Amp^R^; Gm^R^ on mini-Tn*7*; hybrid plasmid consisting of the 4533-bp *Kpn*I/*Bam*HI fragment of pUC18T-mini-Tn*7*T-Gm-rpoD-sfGFP_opt and the 237-bp *Kpn*I/*Bam*HI insert of pEX-A128-Pc promoter	This study
pUC18T-mini-Tn7T-Gm-Pc-sfGFP	Amp^R^; Gm^R^ on mini-Tn*7*; pUC18T-mini-Tn*7*T-Gm-Pc with a 758-bp *Bam*HI/*Sac*I insert containing the sfGFP_opt gene of pUC18T-mini-Tn*7*T-Gm-rpoD-sfGFP_opt	This study
pUC18T-mini-Tn7T-Gm-Pc-mCherry	Amp^R^; Gm^R^ on mini-Tn*7*; pUC18T-mini-Tn*7*T-Gm-Pc with a 749-bp *Bam*HI/*Sac*I insert containing the mCherry_opt gene of pUC18T-mini-Tn*7*T-Gm-rpoD-mCherry_opt	This study
pEX-A258-tdTomato_opt	Amp^R^; pEX-A258 vector with a synthetic 1469-bp *Bam*HI/*Sac*I insert containing the tdTomato gene with optimized codon usage for *Xanthomonadaceae* and fused with an optimized ribosomal binding site of the *rpoD* (SMLT_RS19820) gene of K279a	Eurofins Genomics, Ebersberg, Germany; this study
pUC18T-mini-Tn7T-Gm-Pc-tdTomato	Amp^R^; Gm^R^ on mini-Tn*7*; pUC18T-mini-Tn*7*T-Gm-Pc with a 1469-bp *Bam*HI/*Sac*I insert containing the tdTomato_opt gene of pEX-A258-tdTomato_opt	This study
pEX-A128-mKate2_opt	Amp^R^; pEX-A128 vector with a synthetic 735-bp *Bam*HI/*Sac*I insert containing the mKate2 gene with optimized codon usage for *Xanthomonadaceae* and fused with an optimized ribosomal binding site of the *rpoD* (SMLT_RS19820) gene of K279a	Eurofins Genomics, Ebersberg, Germany; this study
pUC18T-mini-Tn7T-Gm-Pc-mKate2	Amp^R^; Gm^R^ on mini-Tn*7*; pUC18T-mini-Tn*7*T-Gm-Pc with a 735-bp *Bam*HI/*Sac*I insert containing the mKate2_opt gene of pEX-A128-mKate2_opt	This study

### Cell culture.

The Calu-3 human lung adenocarcinoma cell line was obtained from the American Type Culture Collection (ATCC HTB-55, Lot No. 2454196), and all cell culture reagents were purchased from PAN-Biotech (Aidenbach, Germany). The cells were grown in cell culture flasks at 37°C in a humidified atmosphere of 5% CO_2_, using RPMI 1640 culture medium with stable l-glutamine, 2.0 g/L NaHCO_3_ and 10% of heat-inactivated fetal bovine serum (FBS). Adherent Calu-3 cells were dissociated the day before the infection experiments by trypsinization with a trypsin 0.05%/EDTA 0.02% solution at 37°C for 3 min, collected and sedimented by centrifugation at 280 × *g* for 3 min. The cell pellet was then resuspended in culture medium, followed by determination of the cell number with a hemocytometer and evaluation of cell viability with Trypan blue. Finally, the cells were transferred in a volume of 250 μL and a density of 6.25 × 10^4^ cells/cm^2^ into each well of a μ-Slide 8-Well ibiTreat slide (ibidi GmbH, Gräfelfing, Germany).

### Construction of mini-Tn*7* delivery plasmids for fluorescent labeling of S. maltophilia.

Standard recombinant DNA methods were used for nucleic acid preparation and analysis (84). The sequences of primers with integrated recognition sites for restriction enzymes and control primers are listed in Table 3. Using the codon usage adaptation tool Jcat (85), codon optimization of the genes for fluorescent proteins was performed for protein expression in *Xanthomonadaceae*. The successful construction of all plasmids was verified by DNA sequence analysis performed by LGC Biosearch Technologies (Berlin, Germany).

**TABLE 3:** Primers used in this study

Primer	Sequence
KpnI-rpoDprom	ATATggtaccCAATCGATTTGTCGAGATCTGTCGT^*a*^
XhoI-rpoDprom	ATATctcgagTAGTGCTCGCGCCACCG^*b*^
5XhoI-Fluores	ATATctcgagATGGTGAGCAAGGGCGAGG^*b*^
3BamHI-Fluores	ATATggatccTTACTTGTACAGCTCGTCCATGCC^*c*^
5BamHI-Pcprom	TAGATggatccGAATTAAACGGTGGCAGGAGGACTACAT^*c*^
3SacI-Pc-sfGFPopt	TAGATgagctcTTATTACTTGTACAGTTCGTCCATGCCGTGG^*d*^
3SacI-Pc-mChopt	TAGATgagctcTTACTTGTACAGTTCGTCCATGCC^*d*^
PSmlt_glmS-up_	CATCGTCCTTCATCACCACCA
PSmlt_glmS-down_	AAATCTCCTACATCCACGCC
P_Tn_*_7_*_L_	ATTAGCTTACGACGCTACACCC^*e*^
P_Tn_*_7_*_R_	CACAGCATAACTGGACTGATTTC^*e*^

a *Kpn*I site is shown in lower case letters.

b *Xho*I site is shown in lower case letters.

c *Bam*HI site is shown in lower case letters.

d *Sac*I site is shown in lower case letters.

e The primers have been described previously (45).

As a first step, the gene for the red-fluorescence mCherry protein was placed under the control of the *rpoD* promoter of S. maltophilia K279a. The *rpoD* promoter region was obtained by PCR from the genomic DNA of S. maltophilia K279a with primers KpnI-rpoDprom and XhoI-rpoDprom, followed by digestion of the PCR product of 232 bp with *Kpn*l/*Xho*l and cloning into the *Kpn*l/*Xho*l sites of pUC18R6K-mini-Tn7T-Gm to yield pUC18R6K-mini-Tn7T-Gm-rpoD. Then the primer pair 5XhoI-Fluores/3BamHI-Fluores and pGEM-T Easy-mCherry as a template were used to amplify a 731-bp fragment containing the mCherry gene. The PCR product was digested with *Xho*l/*Bam*Hl and ligated into the *Xho*l/*Bam*Hl sites of pUC18R6K-mini-Tn7T-Gm-rpoD, yielding plasmid pUC18R6K-mini-Tn7T-Gm-rpoD-mCherry. To allow for conjugative transfer of the mini-Tn*7* delivery plasmids to S. maltophilia, the pUC18T-mini-Tn7T-Gm-rpoD-mCherry plasmid carrying the *oriT* transfer origin was constructed by ligation of the internal mini-Tn*7* *Kpn*l/*Sa*lI fragment of pUC18R6K-mini-Tn7T-Gm-rpoD-mCherry of 2370 bp with the 3104-bp *Kpn*l/*Sa*lI fragment of pUC18T-mini-Tn7T.

In order to improve protein expression levels in S. maltophilia, the *Apa*l/*Bam*HI fragment within the mini-Tn*7* element of pUC18T-mini-Tn7T-Gm-rpoD-mCherry, comprising the mCherry gene and upstream sequences with the putative ribosomal binding site of *rpoD*, was replaced with *Apa*l/*Bam*HI inserts of pEX-A2-mCherry_opt and pEX-A2-sfGFP_opt to obtain the plasmids pUC18T-mini-Tn7T-Gm-rpoD-mCherry_opt and pUC18T-mini-Tn7T-Gm-rpoD-sfGFP_opt, respectively. The synthetic *Apa*l/*Bam*Hl inserts of pEX-A2-mCherry_opt and pEX-A2-sfGFP_opt were composed of codon-optimized genes for mCherry and the “superfolder” variant of the green fluorescent protein (sfGFP) (86), each fused to an optimized ribosomal binding site of the *rpoD* gene of K279a, respectively.

With the aim of further increasing the rate of gene expression, codon-optimized genes for various fluorescent proteins were placed under the control of the strong, constitutive promoter *P_c_* from class III integron of Delftia acidovorans C17 (62, 63). The synthetic *P_c_* promoter sequence was ligated as a 237-bp *Kpn*l/*Bam*Hl fragment from plasmid pEX-A128-Pc with the 4533-bp *Kpn*l/*Bam*Hl fragment of pUC18T-mini-Tn7T-Gm-rpoD-sfGFP_opt to generate the plasmid pUC18T-mini-Tn7T-Gm-Pc. Next, the mCherry-opt and sfGFP_opt genes were PCR-amplified from pUC18T-mini-Tn7T-Gm-rpoD-mCherry_opt and pUC18T-mini-Tn7T-Gm-rpoD-sfGFP_opt with primer pairs 5BamHI-Pcprom/3SacI-Pc-mChopt and 5BamHI-Pcprom/3SacI-Pc-sfGFPopt, respectively. The putative optimized ribosomal binding site of the *rpoD* gene of K279a was inserted into the PCR products with primer 5BamHI-Pcprom. Finally, the resulting PCR products of 759 bp (mCherry_opt) and 768 bp (sfGFP_opt) were digested with *Bam*Hl/*Sac*l and cloned between the same sites of pUC18T-mini-Tn7T-Gm-Pc to create pUC18T-mini-Tn7T-Gm-Pc-mCherry and pUC18T-mini-Tn*7*T-Gm-Pc-sfGFP, respectively. For construction of pUC18T-mini-Tn7T-Gm-Pc-tdTomato and pUC18T-mini-Tn7T-Gm-Pc-mKate2, the synthetic *Bam*HI/*Sac*l inserts of plasmids pEX-A258-tdTomato_opt (1469 bp) and pEX-A128-mKate2_opt (735 bp) were ligated into the *Bam*HI/*Sac*I sites of pUC18T-mini-Tn7T-Gm-Pc, respectively.

### Delivery of mini-Tn*7* plasmids into S. maltophilia.

The mini-Tn*7* delivery plasmids were transferred to S. maltophilia by four-parental matings, using E. coli DH5α/pRK2013 (87–89) and E. coli SM10 (λ*pir*)/pUX-BF13 (88, 90) as helper strains, and E. coli DH5α/pUC18T-mini-Tn*7*T-Gm-Pc-mCherry, E. coli DH5α/pUC18T-mini-Tn*7*T-Gm-Pc-sfGFP, E. coli DH5α/pUC18T-mini-Tn*7*T-Gm-Pc-tdTomato and E. coli DH5α/pUC18T-mini-Tn*7*T-Gm-Pc-mKate2 as donor strains, respectively. The strains were grown in LB medium supplemented with kanamycin (30 μg/mL) for E. coli DH5α/pRK2013, 100 μg/mL of ampicillin for E. coli SM10 (λ*pir*)/pUX-BF13, and ampicillin (100 μg/mL) and gentamicin (15 μg/mL) for E. coli DH5α strains carrying the mini-Tn*7* plasmids. After overnight growth, the cells from 1 mL of each E. coli culture and 330 μL of S. maltophilia were harvested by centrifugation. To prepare a mating mixture, the cell pellets of all strains were pooled in 1 mL of LB medium, sedimented by centrifugation, resuspended in 100 μL SOB medium and then spotted onto an SOB agar plate as described previously for triparental mating experiments (91). Following overnight incubation of the SOB agar plate at 30°C, the bacterial spot was scraped and resuspended in 1 mL of PBS by rigorous vortexing. The S. maltophilia transconjugants were selected at 37°C on LB agar plates containing 60 μg/mL of gentamicin and 5 μg/mL of norfloxacin to counter-select against the E. coli helper and donor strains.

Chromosomal insertion of the mini-Tn*7* elements in gentamicin-resistant transconjugants was verified by colony PCRs, using the primer pairs P_Tn_*_7_*_L_/PSmlt_glmS-up_ and PSmlt_glmS-down_/P_Tn_*_7_*_R_ (Table 3) to amplify the flanking mini-Tn*7* regions of 671 bp and 502 bp with transposon- and bacterium-specific primers, respectively. For determination of the mini-Tn*7* insertion site, the PCR products were ligated into the pCR2.1 cloning vector of the Original T/A Cloning Kit (ThermoFisher Scientific) and sequenced.

### Confocal laser-scanning microscopy of bacterial cells and biofilms.

Confocal laser-scanning microscopy (CLSM) was used for imaging of bacterial cells and biofilms. To prevent movement during imaging, the bacterial cells were immobilized using the agarose pad method essentially as described previously (92). Briefly, the cells of a 1-mL overnight culture were sedimented by centrifugation, washed once with PBS and resuspended in 250 μL of PBS. The bacterial suspension was then mixed with 1 volume of ProLong Live Antifade Reagent in PBS (ThermoFisher Scientific) and placed between a coverslip and a thin pad of 0.5% agarose in PBS.

To prepare the inoculum for biofilm formation on an abiotic polymer surface, exponentially growing cultures of fluorescently labeled strains were adjusted to a cell number of about 1.43 × 10^4^ CFU/mL in 0.5 × Brain Heart Infusion (BHI) broth. Each well of a μ-Slide 8-Well ibiTreat slide was then seeded with a total volume of 350 μL, containing about 5 × 10^3^ CFU of each bacterium, followed by incubation of the μ-Slides at 30°C without shaking in a humidified chamber. Exhausted medium was replaced by 350 μL of fresh, prewarmed 0.5 × BHI broth every 8 to 16 h. After an incubation for 48 or 72 h, the culture supernatants were aspirated and the wells were washed with 350 μL of PBS. The biofilms were then fixed in the dark with 350 μL of a 2% paraformaldehyde (PFA) solution in PBS at room temperature for 20 min. Finally, the biofilms were washed with 350 μL of PBS and overlaid with 200 μL of ProLong Live Antifade Reagent in PBS as recommended by the supplier (ThermoFisher Scientific).

For biofilm experiments on a biotic surface, only Calu-3 cells that reached a confluence of at least 90% were used for infection with S. maltophilia Sm314::sfGFP cells of the early exponential growth phase. The multiplicity of infection was adjusted to 10. Following incubation of the μ-Slides at 37°C in a humidified atmosphere of 5% CO_2_ for 16, 24 and 48 h, samples were fixed in the dark with 300 μL of a 1% PFA solution in PBS at room temperature for 20 min. The samples were then washed twice with 200 μL of PBS for 5 min each. Permeabilization of fixed cells was achieved by treatment of the samples with 150 μL of 0.5% Triton X-100 in PBS at room temperature for 10 min. The samples were washed three times with 200 μL of PBS for 5 min each and then blocked with 200 μL of 5% FBS in PBS at room temperature for 30 min. Staining of Calu-3 cells with Alexa Fluor 647 anti-cytokeratin (pan reactive) antibody (clone C-11; BioLegend) at a final concentration of 1 μg/mL was performed in 150 μL of 5% FBS in PBS containing 200 U/mL penicillin and 200 μg/mL streptomycin. After overnight incubation at 4°C, samples were washed three times with 200 μL of PBS for 5 min each and incubated in the dark with 150 μL of 300 nM DAPI (BioLegend) at room temperature for 10 min. Following an additional wash step with 200 μL of PBS, samples were overlaid with 200 μL of ProLong Live Antifade Reagent.

Imaging was performed with a TCS SP5 inverse confocal laser-scanning microscope (Leica Microsystems, Mannheim, Germany) and analyzed with LAS AF software (version 2.73). The microscope was equipped with a Leica 63x/NA 1.40 HCX Plan Apochromat CS oil immersion objective. The sfGFP protein was excited with 488 nm laser light, and fluorescence emission was detected between 495 and 530 nm, whereas mKate2 was excited with laser light at 594 nm and fluorescence emission was detected between 605 and 670 nm. The excitation wavelength of laser light was 561 nm for both mCherry and tdTomato, but emission of their fluorescence was detected between 573 and 629 nm and 580 and 648, respectively. The excitation wavelength of laser light was 405 nm for DAPI, and its fluorescence emission was detected between 415 and 470 nm. Alexa Fluor 647 was excited with laser light at 633 nm, and fluorescence emission was detected between 660 and 685 nm. Three-dimensional biofilm images were routinely generated from confocal image stacks using the *daime* computer program (93). However, in some specific cases, the confocal image stacks were further processed with Leica's Lightning Deconvolution Tool (Leica Application Suite X, version 3.0.0_15697) and the Imaris Viewer 10.0.0 (Oxford Instruments).

### Stability testing.

The genome stability of mini-Tn*7* insertions was investigated after growth of the bacteria in the absence of antibiotic selection pressure for 5 days essentially as described previously (44, 94). To ensure that the initial cultures have retained their selection marker at the beginning of the experiment, fluorescently labeled strains were grown aerobically (220 rpm) at 37°C overnight in LB medium containing 60 μg/mL gentamicin. From the overnight cultures, each strain was streaked onto LB agar with 60 μg/mL gentamicin to grow single colonies at 37°C as controls for later colony PCRs. The initial overnight cultures were then grown for five consecutive days at 37°C on a rotary shaker at 200 rpm, with daily dilutions of the cultures by a thousandfold into fresh LB medium. Finally, serial dilutions of the cultures were plated on LB agar without gentamicin to subsequently test 100 single colonies of each strain for their growth on LB agar containing 60 μg/mL gentamicin. The presence and the correct orientation of the mini-Tn*7* elements was examined in 10 randomly selected colonies of each strain by colony PCRs with primer pair PSmlt_glmS-down_/P_Tn_*_7_*_R_ (Table 3).

### Growth of strains with mini-Tn*7* insertions.

To compare the growth of fluorescently labeled strains with that of their parental strains, fresh overnight cultures were adjusted to an OD_600_ of 0.1 with LB-Miller medium, followed by transfer of 100 μL of each diluted culture to 100 μL of LB-Miller medium in each well of a Honeycomb X100 Bioscreen C sterile plate (ThermoFisher Scientific). The growth at 37°C and 220 rpm was recorded by measurement of the OD_600_ values at 15-minutes intervals for a total of 24 h using a Labsystems Bioscreen C automated microbiology growth curve analysis system (Labsystems, Helsinki, Finland). All growth curves were plotted in triplicate. Based on graphs of log OD_600_ versus time and estimation of the bacterial cell number from optical density, the generation time (G) of a strain was calculated using the formula G = t/(3.3 log N_t_/N_0_), where t was the time interval, N_t_ the bacterial cell number at the end of the time interval and N_0_ the bacterial cell number at the beginning of the time interval.

### Antibiotic susceptibility testing.

MICs were determined by the broth microdilution method in cation-adjusted Mueller-Hinton broth (CAMHB) following the recommendations of the Clinical and Laboratory Standards Institute (CLSI) (75). The antibiotic stock solutions were prepared according to the CLSI guidelines and the test panel included 18 antibiotics of different classes. Fluorescently labeled and wild-type parental strains were first grown overnight in CAMHB under the conditions recommended by the CLSI. The MICs were determined in sterile 96-well microplates using 2-fold serial dilutions of each antibiotic. After overnight growth of the strains, 100 μL of diluted bacterial cultures with a cell number of about 5 × 10^5^ CFU/mL each were added to the wells containing the serial antibiotic dilutions. The microtiter plates were analyzed after 20 h of incubation at 37°C. The MIC was defined as the lowest concentration of the antibiotic (in μg/mL) that prevented visual growth. MIC determinations were done in duplicate.

### Biofilm formation assay.

To evaluate biofilm formation, overnight cultures of the strains were grown aerobically (200 rpm) in 0.5 × BHI broth at 37°C, followed by dilution of each culture with fresh medium to an optical density at 620 nm (OD_620_) of 0.05. Sterile, untreated 96-well microtiter plates (BrandTech 781662) were then inoculated with the bacterial suspensions (200 μL per well) and incubated at 37°C for 24 h. Prior to biofilm quantification, cell growth was estimated in each well by measuring the OD_620_ value using a microplate reader (Multilabel Plater Reader VICTOR^3^, PerkinElmer). Quantification of the biofilm biomass was performed by crystal violet (CV) staining as described previously (95). The amount of biofilm was quantified by measuring the OD_550_ of dissolved CV using the microplate reader. Biofilm formation (OD_550_ of CV) was normalized by cell growth (OD_620_) and reported as relative biofilm formation. For each strain, four biological replicates with six technical replicates each were prepared. Statistical significance was determined by the non-parametric Kruskal-Wallis one-way Analysis of Variance (ANOVA) test corrected for multiple comparisons using Dunn’s test.

### Virulence in Galleria mellonella.

Larvae of Galleria mellonella were reared in-house. A total of 30 larvae with a weight between 300 and 400 mg and no signs of melanization were infected with each S. maltophilia strain. For preparation of bacterial inoculums, fluorescently tagged and corresponding parental wild-type strains were grown overnight in 10 mL of BHI medium at 37°C on a rotary shaker at 200 rpm. The bacterial cells were then sedimented by centrifugation, washed in PBS and adjusted to contain about 5 × 10^5^ cells per larva, which has been shown previously as an optimal dose of S. maltophilia required to kill G. mellonella over a period of 24 to 96 h (38, 96). The bacterial burden of the doses was confirmed by plating the cells onto BHI agar. The larvae were infected with 10 μL of the inoculum through the left proleg using a 50 μL-Hamilton Microliter syringe and incubated in the dark at 30°C in empty Petri dishes. Survival of infected larvae was scored at 24-h intervals for 5 days. The larvae were considered dead when they failed to respond to touch, which was equivalent to complete melanization (blackening of the larvae). At least two replicates were performed with different batches of larvae. Kaplan–Meier survival curves were plotted using GraphPad Prism 5.0a, and survival analysis and statistical significance was determined using the log-rank test (GraphPad Software, San Diego, CA).

### Data availability.

The complete nucleotide sequences of delivery plasmids pUC18T-mini-Tn7T-Gm-Pc-mCherry (Addgene plasmid # 199247), pUC18T-mini-Tn7T-Gm-Pc-sfGFP (Addgene plasmid # 199248), pUC18T-mini-Tn7T-Gm-Pc-mKate2 (Addgene plasmid # 199249) and pUC18T-mini-Tn7T-Gm-Pc-tdTomato (Addgene plasmid # 199250) have been deposited in the GenBank database under accession numbers. OP566392, OP566393, OP566394 and OP566395, respectively.

The plasmids are available through the Addgene repository upon request.
