# Supplementary material for: Improved mini-Tn7 Delivery Plasmids for Fluorescent Labeling of Stenotrophomonas maltophilia
Source: Appl Environ Microbiol. 2023 May 17;89(6):e00317-23. doi: 10.1128/aem.00317-23 (PMC10304964; doi:10.1128/aem.00317-23)
Supplement: Supplemental file 1 — Supplemental material. Download aem.00317-23-s0001.docx, DOCX file, 3.4 MB [file aem.00317-23-s0001.docx]

**SUPPLEMENTAL MATERIAL FOR**

Improved mini-Tn*7* delivery plasmids for fluorescent labelling of *Stenotrophomonas maltophilia*

Uwe Mamat,^a^# Manuel Hein,^a^ Dörte Grella,^a^ Claire S. Taylor,^a^ Thomas Scholzen,^b^ Ifey Alio,^c^ Wolfgang R. Streit,^c^ Pol Huedo,^d,e^ Xavier Coves,^d,e^ Oscar Conchillo-Solé,^d,e^ Andromeda-Celeste Gómez,^d,e^ Isidre Gibert,^d,e^ Daniel Yero,^d,e^ Ulrich E. Schaible^a^

^a^Cellular Microbiology, Priority Research Area Infections, Research Center Borstel, Leibniz Lung Center, Borstel, Germany

^b^Core Facility Fluorescence Cytometry, Research Center Borstel, Leibniz Lung Center, Borstel, Germany

^c^Department of Microbiology and Biotechnology, Universität Hamburg, Hamburg, Germany

^d^Institut de Biotecnologia i de Biomedicina (IBB), Universitat Autònoma de Barcelona (UAB), Barcelona, Spain

^e^Departament de Genètica i de Microbiologia, Universitat Autònoma de Barcelona (UAB), Barcelona, Spain

Running Head: mini-Tn*7* delivery plasmids for *Stenotrophomonas*

#Address correspondence to Uwe Mamat, umamat@fz-borstel.de

Keywords: *Stenotrophomonas maltophilia*, fluorescent labelling, transposon, mini-Tn*7*, biofilm


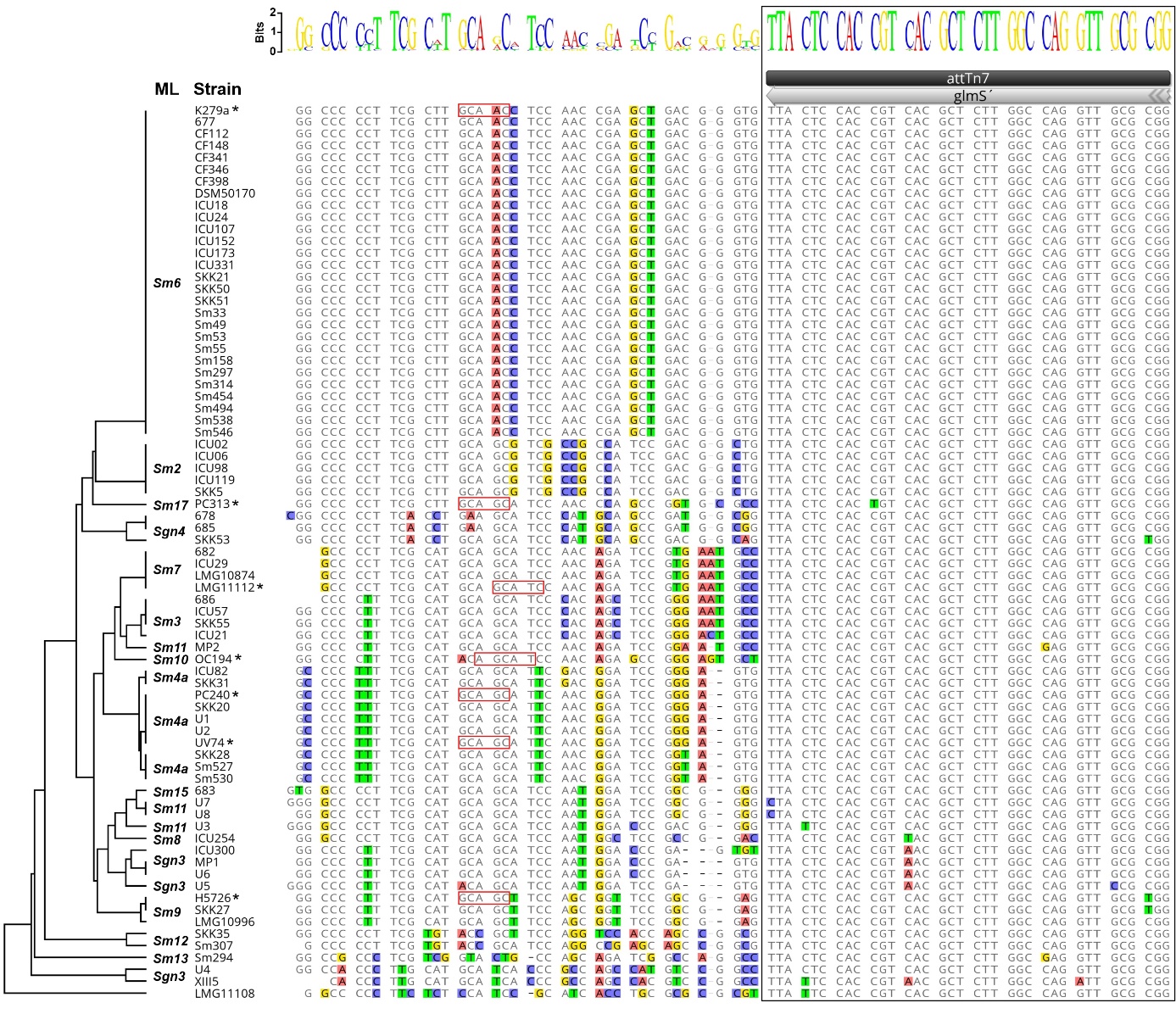
**SUPPLEMENTAL FIGURE S1**

**Figure S1** Alignment of *att*Tn*7*-associated sequence motifs at the immediate 3´-end of the *glmS* gene (framed sequences) and downstream sequences of the intergenic region of 75 *S. maltophilia* isolates of different monophyletic lineages (ML). The mini-Tn*7* insertion sites were determined for strains K279a, PC313, LMG11112, OC194, PC240, UV74 and H5726 (marked with asterisks) and are shown in red boxes (see Figure 2 of the main text). The alignment was created with Geneious Prime^®^ 2023.0.4 ([www.geneious.com](file:///C:\Users\uwema\Documents\Manuscript%20construction%20of%20fluorescently%20tagged%20strains\www.geneious.com)).

**SUPPLEMENTAL FIGURE S2**

**
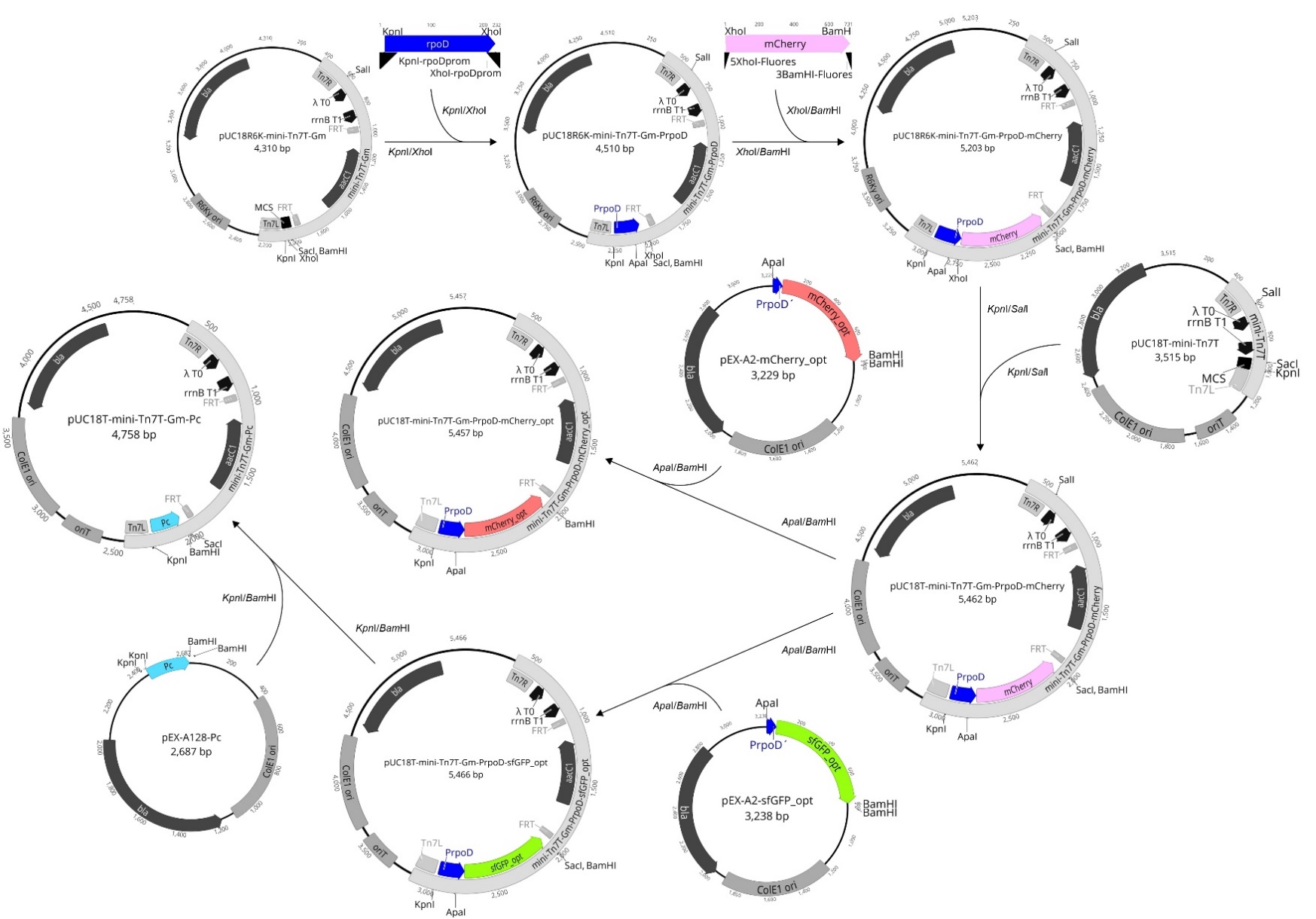
**

**SUPPLEMENTAL FIGURE S2, continued**

**
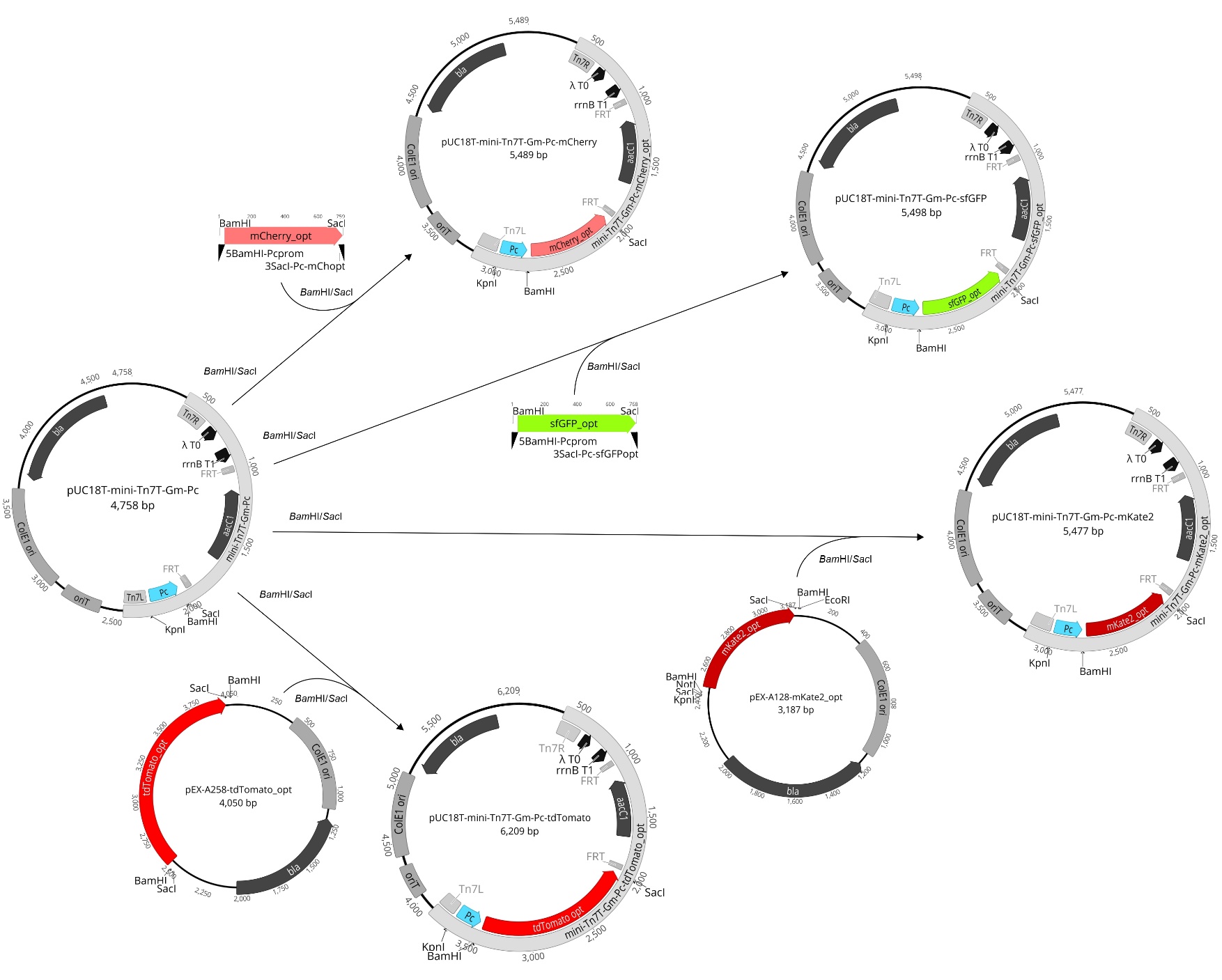
**

**Figure S2** Workflow for the construction of the mini-Tn*7* delivery plasmids pUC18T-mini-Tn7T-Gm-Pc-sfGFP, pUC18T-mini-Tn7T-Gm-Pc-mCherry, pUC18T-mini-Tn7T-Gm-Pc-tdTomato and pUC18T-mini-Tn7T-Gm-Pc-mKate2 for fluorescent labelling of *S. maltophilia*. The plasmids pUC18R6K-mini-Tn7T-Gm and pUC18T-mini-Tn7T (1, 2) served as the base vectors. For details of the cloning strategy, see the Materials and Methods section. The plasmid maps were created with Geneious Prime^®^ 2023.0.4 ([www.geneious.com](file:///C:\Users\uwema\Documents\Manuscript%20construction%20of%20fluorescently%20tagged%20strains\www.geneious.com)). Not drawn to scale.

**SUPPLEMENTAL FIGURE S3**


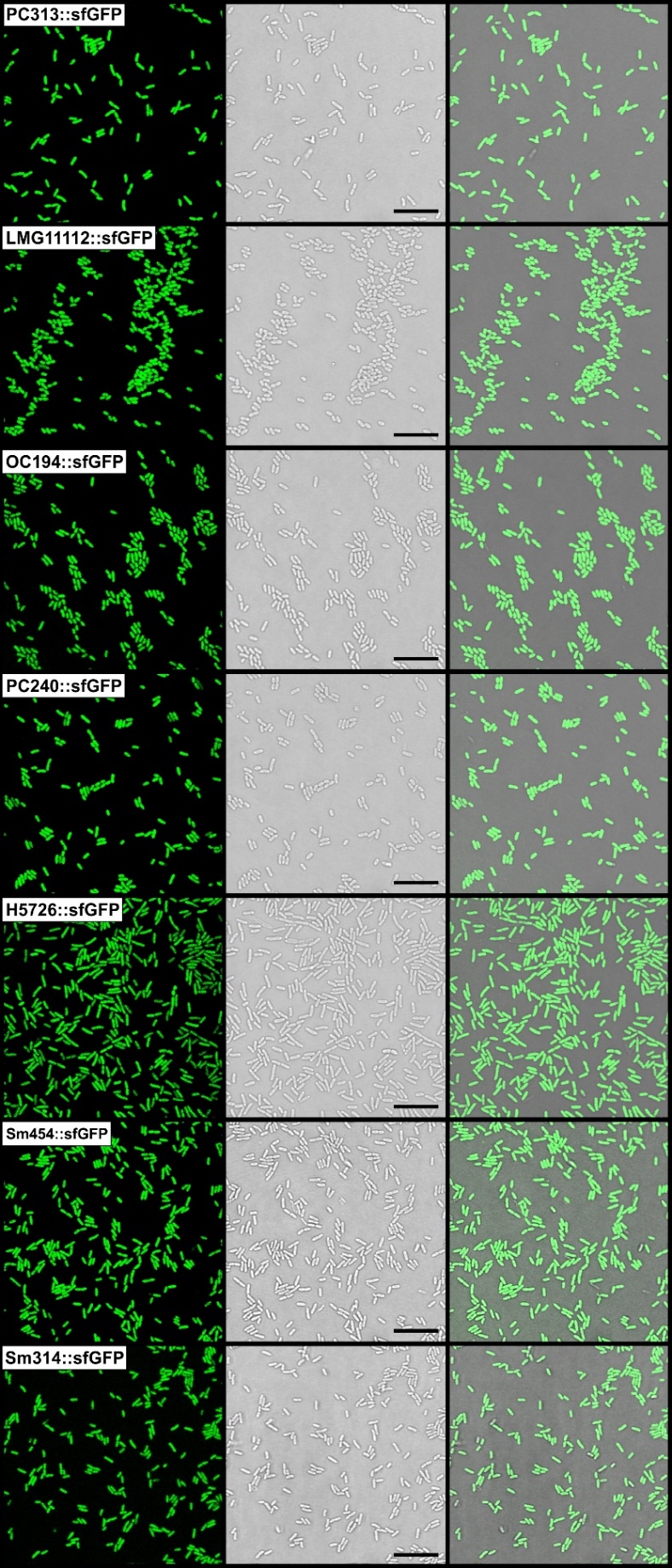


**Figure S3** Confocal (left panel), transmitted light (middle panel) and merged (right panel) images of *S. maltophilia* strains carrying the chromosomally integrated mini-Tn*7*T-Gm-Pc-sfGFP_opt element for expression of sfGFP. The images indicate that all cells of each strain are fluorescently labelled. Scale bars correspond to 10 µm.


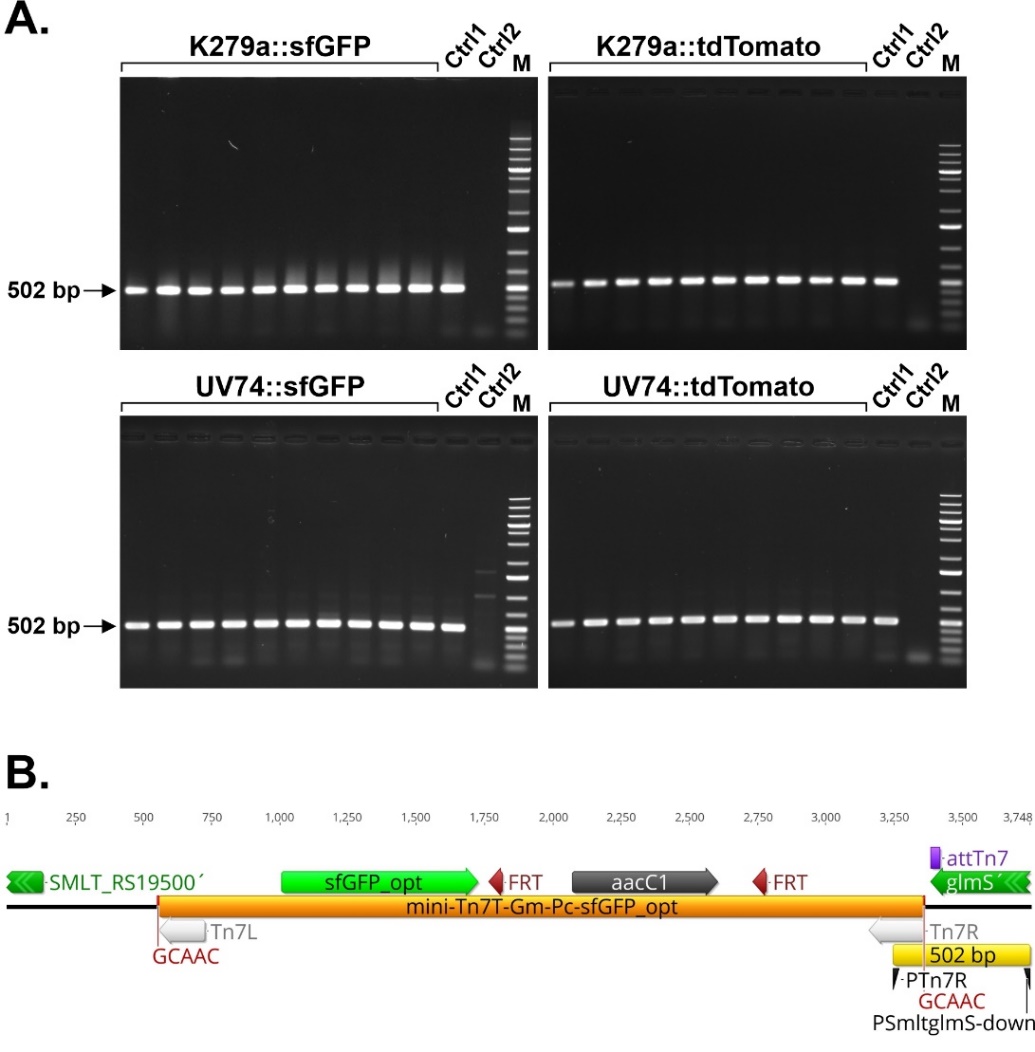
**SUPPLEMENTAL FIGURE S4**

**Figure S4**  Stability of mini-Tn*7* insertions in fluorescently labelled *S. maltophilia* strains K279a::sfGFP, K279a::tdTomato, UV74::sfGFP and UV74::tdTomato after growth of the strains in the absence of gentamycin selection for five days. (A.) The presence of the mini-Tn*7* elements in the correct orientation was confirmed by PCR using 10 randomly selected clones of each strain as templates and primer pair PSmlt_glmS-down_/P_Tn_*_7_*_R_ (see the Material and Methods section for details). Detection of the 502-bp amplification products by electrophoresis in 0.8% agarose gels indicated the presence of the mini-Tn*7* elements. The positive control lane Ctrl1 and the negative control lane Ctrl2 contained the PCR products of the fluorescently labelled strains and their corresponding parents, respectively, whereas lane M contained the Generuler 1 kb Plus DNA Ladder (Thermo Fisher Scientific) as a molecular mass marker. (B.) The genetic map shows the mini-Tn*7*T-Gm-Pc-sfGFP_opt element inserted in the intergenic region between the genes *glmS* and *smlt4098* (SMLT_RS19500) of *S. maltophilia* K279a::sfGFP, including the location of the control primer pair PSmlt_glmS-down_/P_Tn_*_7_*_R_ at the 3´-end of the *glmS* gene and within the Tn*7*R end of the transposon, respectively. Also shown is the GCAAC target sequence of Tn*7* in *S. maltophilia* K279a, which is duplicated at the ends of the mini-Tn*7* element as a result of the transposition event. The map was generated with Geneious Prime^®^ 2023.0.4 ([www.geneious.com](file:///C:\Users\uwema\Documents\Manuscript%20construction%20of%20fluorescently%20tagged%20strains\www.geneious.com)).

**SUPPLEMENTAL FIGURE S5**


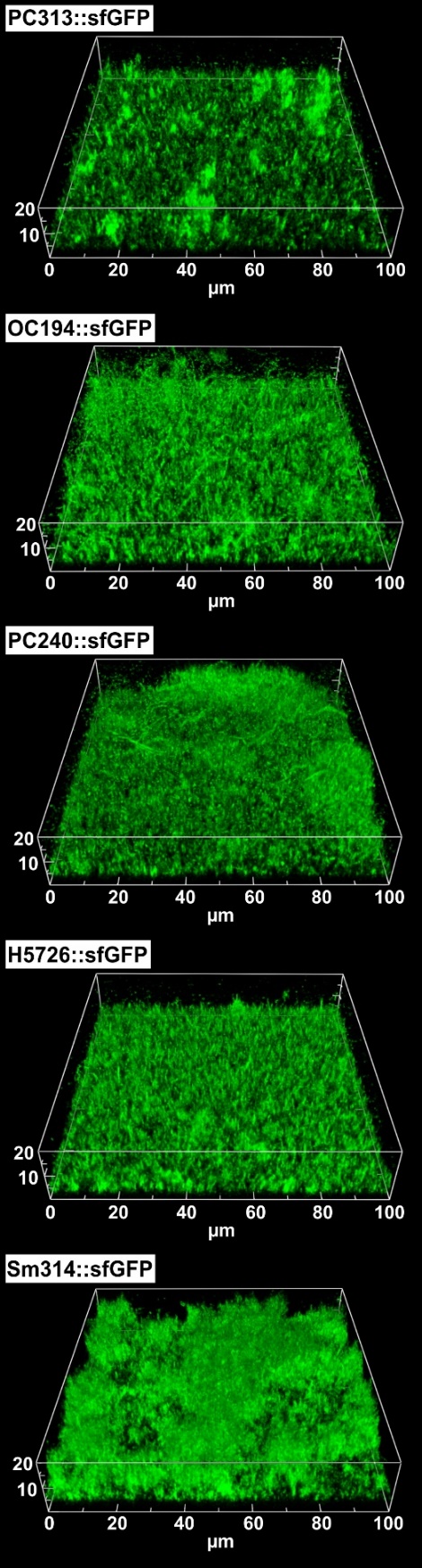


**Figure S5**  Representative confocal laser-scanning microscopy (CLSM) images showing the architecture of biofilms of different sfGFP-labelled *S. maltophilia* strains grown on a polymer surface for 72 hours. The three-dimensional images were generated with *daime* (3).

**SUPPLEMENTAL FIGURE S6**


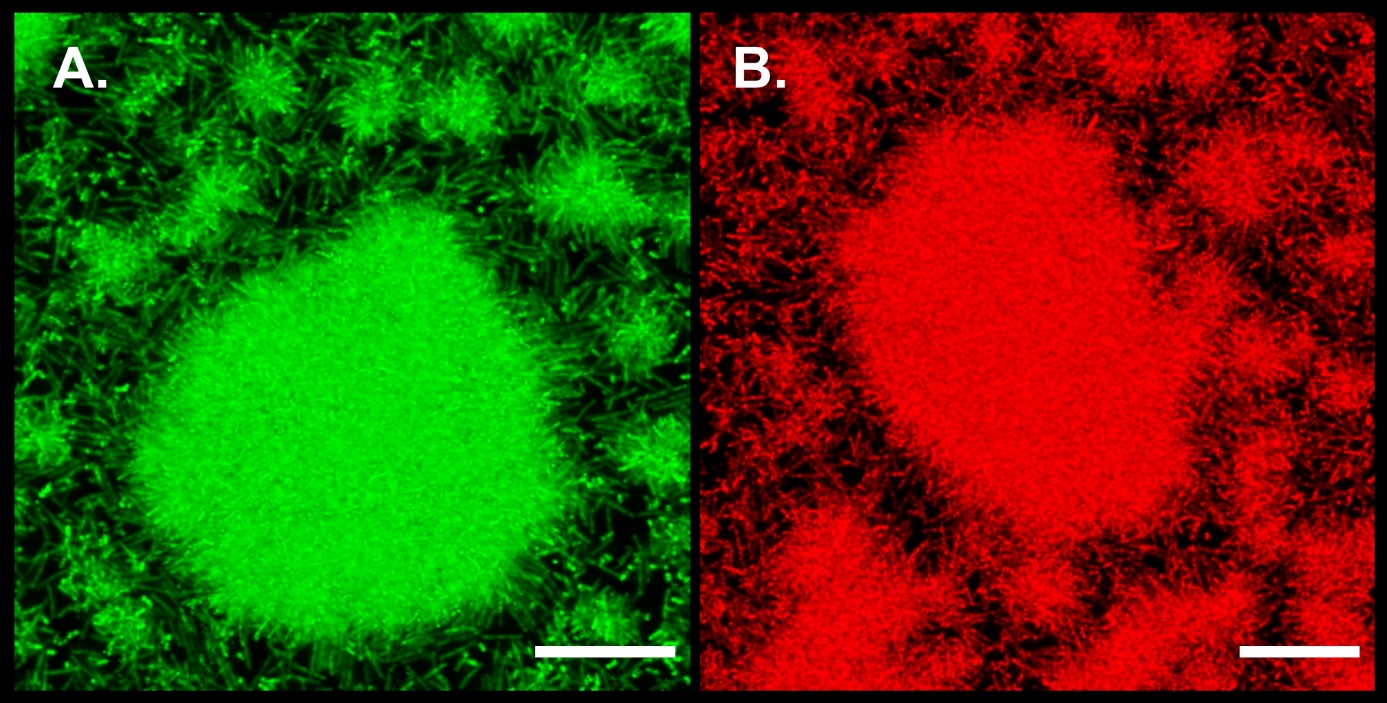


**Figure S6**  CLSM images of biofilm microcolonies of *S. maltophilia* strains Sm454::sfGFP (A.) and Sm454::mKate2 (B.) grown on a polymer surface for 72 hours. The confocal image stacks were processed with Leica's Lightning Deconvolution Tool (Leica Application Suite X, version 3.0.0_15697) and the Imaris Viewer 10.0.0 (Oxford Instruments). Scale bars correspond to 5 µm.

**SUPPLEMENTAL FIGURE S7**


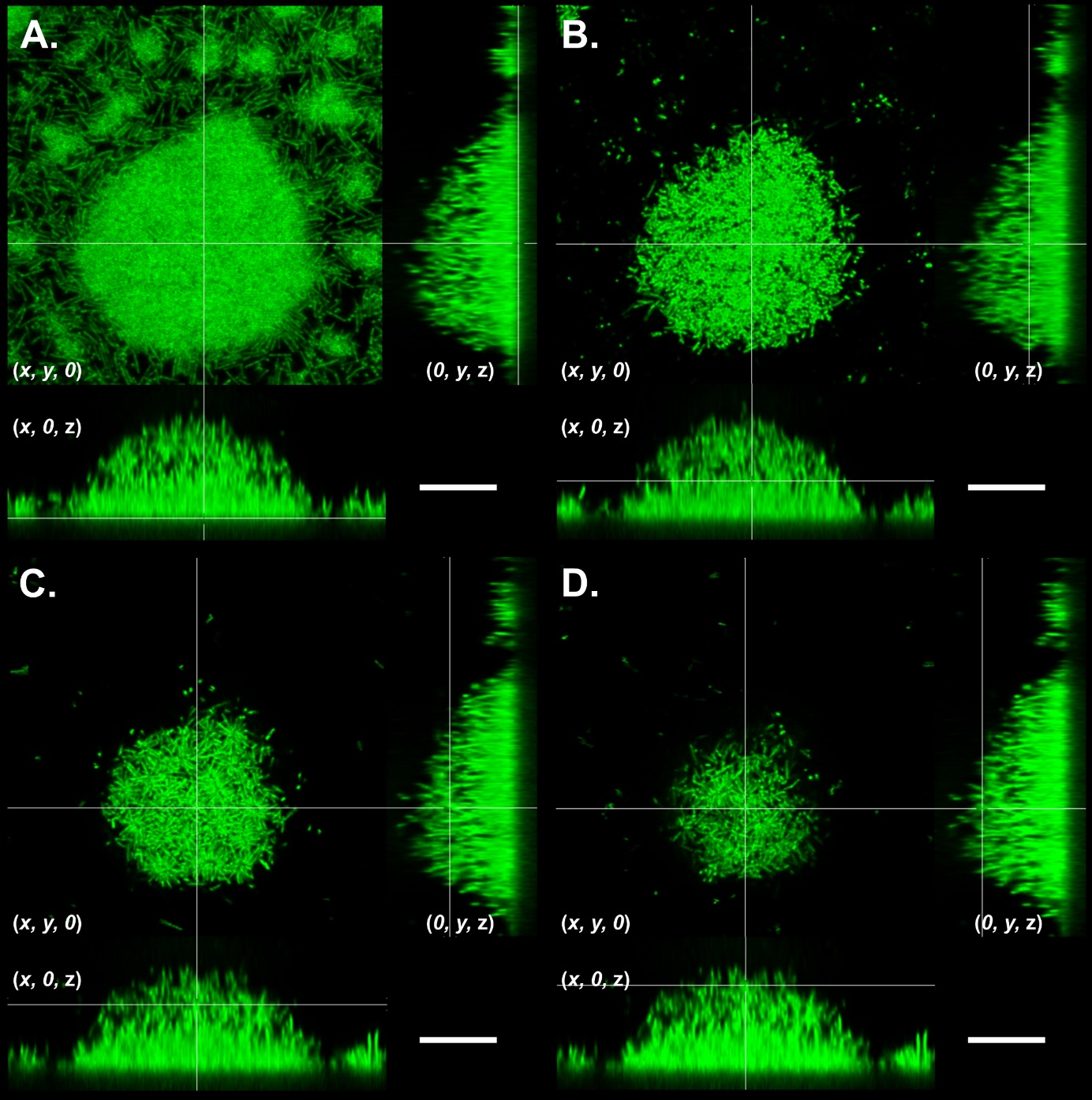


**Figure S7** Dissection of a biofilm microcolony of *S. maltophilia* strain Sm454::sfGFP grown on a polymer surface for 72 hours. Orthogonal projections of confocal z-axis slice sections indicated that densely packed cell layers were localized at the bottom and middle of the microcolony (A. and B.), whereas layers of loosely packed rosette-shaped cell aggregates formed the upper part of the microcolony (C. and D.). The confocal image stacks were processed with Leica's Lightning Deconvolution Tool (Leica Application Suite X, version 3.0.0_15697) and the Imaris Viewer 10.0.0 (Oxford Instruments). Scale bars correspond to 10 µm.

**REFERENCES**

1. Choi KH, DeShazer D, Schweizer HP. 2006. mini-Tn*7* insertion in bacteria with multiple *glmS*-linked attTn*7* sites: example *Burkholderia mallei* ATCC 23344. Nat Protoc 1:162-169.

2. Choi KH, Gaynor JB, White KG, Lopez C, Bosio CM, Karkhoff-Schweizer RR, Schweizer HP. 2005. A Tn*7*-based broad-range bacterial cloning and expression system. Nat Methods 2:443-448.

3. Daims H, Lucker S, Wagner M. 2006. *daime*, a novel image analysis program for microbial ecology and biofilm research. Environ Microbiol 8:200-213.
